# Misregulation of the *IgH* Locus in Thymocytes

**DOI:** 10.3389/fimmu.2018.02426

**Published:** 2018-11-13

**Authors:** Gita Kumari, Tatiana Gerasimova, Hansen Du, Supriyo De, William H. Wood, Kevin G. Becker, Ranjan Sen

**Affiliations:** ^1^Laboratory of Molecular Biology and Immunology, National Institute on Aging, Baltimore, MD, United States; ^2^Laboratory of Genetics and Genomics, National Institute on Aging, Baltimore, MD, United States

**Keywords:** VDJ recombination, *IgH* locus, thymocytes, enhancer, chromatin, B cells

## Abstract

Functional antigen receptor genes are assembled by somatic rearrangements that are largely lymphocyte lineage specific. The immunoglobulin heavy chain (*IgH*) gene locus is unique amongst the seven antigen receptor loci in undergoing partial gene rearrangements in the wrong lineage. Here we demonstrate that breakdown of lineage-specificity is associated with inappropriate activation of the Eμ enhancer during T cell development by a different constellation of transcription factors than those used in developing B cells. This is reflected in reduced enhancer-induced epigenetic changes, eRNAs, formation of the RAG1/2-rich recombination center, attenuated chromatin looping and markedly different utilization of D_H_ gene segments in CD4^+^CD8^+^ (DP) thymocytes. Additionally, CTCF-dependent V_H_ locus compaction is disrupted in DP cells despite comparable transcription factor binding in both lineages. These observations identify multiple mechanisms that contribute to lineage-specific antigen receptor gene assembly.

## Introduction

Somatic gene rearrangements assemble B and T cell antigen receptor genes from individual gene segments during lymphocyte development. This process of V(D)J recombination is lineage specific, such that T cell receptor (TCR) genes recombine only in T lineage cells and immunoglobulin genes recombine completely only in B lineage cells. The only exception to this rule is that the immunoglobulin heavy chain (*IgH*) gene locus undergoes partial rearrangements in the T lineage (1, 2). Functional *IgH* genes are assembled by two recombination events. The first juxtaposes a diversity (D_H_) gene segment to a joining (J_H_) gene segment, and the second recombines a variable (V_H_) gene segment to the pre-assembled DJ_H_ junction (3, 4). D_H_ rearrangements have been shown to occur at several stages of T cell development, including the double positive (CD4^+^CD8^+^) stage and ~50% of mature T cells have a DJ_H_ rearranged *IgH* allele (2, 5). V_H_ recombination has never been detected in WT thymocytes. Several forms of genetic manipulation can, however, induce restricted V_H_ recombination in DP thymocytes. For example, forced expression of Pax5 or inactivating the intergenic control region 1 (IGCR1) leads to recombination of D_H_-proximal V_H_7183 gene segments (6–8). Additionally, introduction of a V_H_ gene segment near DFL16.1 results in its recombination in DP cells (9). The breakdown of lineage specificity of *IgH* locus rearrangements remains a unique feature amongst antigen receptor genes. Our working hypothesis is that understanding this phenomenon may provide insight into regulatory mechanisms that impose specificity of V(D)J recombination and more generally into tissue-specific gene expression.

Recombination activating gene products Rag1 and Rag2 initiate V(D)J recombination at immunoglobulin and TCR loci by introducing double-strand breaks at recombination signal sequences (RSSs) associated with rearrangeable gene segments (10, 11). Accessibility of the recombinase to antigen receptor loci is governed by regulated changes in chromatin structure of individual V, D, and J gene segments. This is referred to as the chromatin accessibility hypothesis which originates from observations that activation for rearrangement correlates with transcription of unrearranged loci (12, 13). Subsequent studies showed that transcriptional enhancers associated with antigen receptor loci are required for lineage-specific V(D)J recombination (14–19). Thus, enhancers are at the crux of the accessibility hypothesis.

Several studies demonstrate that breakdown of lineage-specific recombination at the *IgH* locus is related to enhancer activity. Ferrier et al. first showed that *IgH* intronic enhancer Eμ supports TCR Dβ to Jβ recombination on a transgenic substrate in both T cells and B cells (20). These observations were extended by replacement of TCRβ enhancer (Eβ) with Eμ at TCRβ locus that permitted partial Dβ to Jβ rearrangements in T cells (14). Conversely, Afshar et al. reported that Eμ deletion at the *IgH* locus abrogated D_H_ to J_H_ recombination in thymocytes (21). Since Eμ is essential for efficient V(D)J recombination in pro-B cells, these observations suggest that lack of lineage specificity of Eμ underlies promiscuous D_H_ recombination in DP thymocytes. However, the extent and basis of Eμ activity in DP thymocytes has not been addressed.

To better understand the mechanisms of partial *IgH* rearrangements in thymocytes, we examined transcription, recombination and epigenetic state of the *IgH* locus in CD4^+^CD8^+^ (DP) thymocytes. We found the locus to be partially active in DP cells compared to pro-B cells by all criteria assayed. This state correlated with the absence of a subset of transcription factors from Eμ in DP thymocytes compared to pro-B cells, suggesting that partial locus activation resulted from inappropriate Eμ function. We also found that CTCF-dependent steps of *IgH* locus compaction were abrogated in DP thymocytes despite binding of this architectural protein throughout the locus, providing a plausible explanation for the lack of V_H_ recombination in these cells. Our observations highlight lineage-specific steps of locus activation that are required for complete *IgH* gene rearrangements in pro-B cells.

## Materials and methods

### Cell purification

CD19^+^ pro-B cells were purified from Rag2^−/−^ C57BL/6 mice by positive selection using CD19 beads (Stem Cell Technology, Cat # 18754). CD4^+^CD8^+^ cells mice were purified from thymii of TCRβ × Rag2^−/−^ transgenic mice by positive selection using CD8 beads per manufacturer's instruction (Stem Cell Technology Cat # 18753). All mouse experiments were done in accordance with Animal Care and Use Committee of the National Institute on Aging.

WT pro-B cells were purified from 8- to 10-week-old C57BL/6 mice. Bone marrow cells from C57BL/6 mice were depleted of macrophages, granulocytes, erythroid lineage and T cells using biotinylated antibodies against CD11b (Cat # 553309), Gr-1 (Cat # 553125), Ter119 (Cat # 553672), CD3ε (Cat # 553060), and Ly-6C (Cat # 557359) from BD Biosciences followed by staining with PE-Cy7 conjugated streptavidin (Biolegend, Cat # 557598). IgM expressing cells were then removed using FITC-conjugated affini pure Fab fragment goat anti-mouse IgM μ chain specific antibody (Jackson Immuno Research, Cat # 115097029). Thereafter, pro-B cells were sorted as IgM^−^ B220^+^ CD43^+^ CD19^+^ AA4.1^+^ on a BD FACS Aria Fusion. BV421-anti-B220 (Biolegend, Cat # 103240), PE-anti-CD43 (BD Biosciences, Cat # 553271), APC-anti-AA4.1 (eBioscience, Cat # 136510), APC-Cy7-anti-CD19 (Biolegend, Cat # 115529) were used for labeling.

WT DP thymocytes were enriched from thymii of C57BL/6 mice by positive selection using CD8 beads per manufacturer's instruction (Stem Cell Technology, Cat # 18753).

### Chromatin immunoprecipitation (ChIP)

Chromatin immunoprecipitation of modified histones and transcription factors were carried out with pro-B cells derived from bone marrow of Rag2^−/−^ mice and DP thymocytes derived from TCRβ × Rag2^−/−^ transgenic mice or WT mice as described previously (22). Modified histone antibodies were purchased from Active Motif: anti-H3K4me3 (Cat # 39519), anti-H3K9ac (Cat # 39137), anti-H3K27me3 (Cat # 39155). Antibodies for transcription factors were as follows: anti-E2A (Cat # Sc-349), anti-YY1 (Cat # Sc-1703), anti-Ets-1 (Cat # Sc-350), Anti-HEB (Cat # Sc-357) were from Santa Cruz Biotechnology and anti-Runx1 (Cat # ab23980), anti-Rad21 (Cat # ab992) were from Abcam; anti-CTCF (Cat # 07-729) was purchased from Millipore. Formaldehyde cross-linked and sonicated chromatin from 5 × 10^6^ cells was pre-cleared with 5 μg of non-specific rabbit IgG and immunoprecipitated with the relevant antibody or an equal amount of nonspecific IgG. The coprecipitated DNA was purified and analyzed by real-time PCR. Input DNA and the immunoprecipitated DNA were quantified using PicoGreen (Molecular Probes/Life Technologies). For analysis of enrichment, 200 pg of DNA was used in each real-time PCR reaction performed in triplicate and each ChIP was performed in duplicate. The relative abundance of amplicons in the immunoprecipitated DNA relative to input was analyzed by real-time PCR using the primers listed in Table S1. Rag1/Rag2 ChIP was carried out using anti-Rag1 (Abcam, Cat # ab172637) and anti-Rag2 (David Schatz, Yale University) antibodies as described by Ji et al. (23).

### DNase I sensitivity assay

10^7^ nuclei from Rag2^−/−^ CD19^+^ cells and CD4^+^CD8^+^ cells from TCRβ × Rag2^−/−^ transgenic mice were treated with different concentrations of DNase I. Twenty-five nanograms of purified genomic DNA was used in quantitative PCR assays performed in duplicate with primer pairs shown in Table S1. The amplicons were normalized to the amount of intact β-globin alleles at each DNase I concentration as described previously (24).

### RNA analysis

Total RNA was extracted from Rag2^−/−^ CD19^+^ cells and CD4^+^CD8^+^ cells derived from TCRβ × Rag2^−/−^ transgenic mice using RNeasy plus microkit (Qiagen). Two hundred nanograms of RNA was reverse transcribed with Superscript III (Invitrogen) and strand-specific primers were used according to manufacturer's protocol. Quantitative PCR was performed with SYBR green using primer pairs described in Table S1.

For analysis of Eμ-sense and antisense transcripts, strand specific primers were used to prime cDNA synthesis and amplified amplicons were used for analysis of copy number of sense and antisense RNA. To calculate the number of eRNA molecules, a standard curve was generated by plotting the Ct values of known concentration of 1 kb DNA (amplified from genomic DNA) which covers both sense and antisense transcribed region of *IgH* locus. The copy number of sense and antisense RNA was calculated (after RT and qPCR) using the equation (Copy number = amount of cDNA in femtogram ^*^ 6.022 × 10^23^ / length of cDNA in base pairs ^*^ 1 × 10^15^^*^ 650).

### D_H_ rearrangements

Fifty nanograms of genomic DNA and 4-fold serial dilutions from pro-B cells and CD4^+^CD8^+^ cells from wild type C57BL/6 mice were used to amplify DJ_H_ junctions using primers listed in Table S1. An amplicon from the mouse β-globin locus was used to normalize across samples.

For deep sequencing, DSP2-J_H_1 amplified products were ligated to adaptors and sequenced using Ion Proton sequencer (ThermoFisher Scientific). FASTQ files containing single-end, variable length reads were obtained from the Ion Proton sequencer. Adaptor contamination and low-quality bases (below FRED quality score of 20) were removed by Cutadapt program leaving reads more than 160 bases long for further analysis. Duplicate reads from FASTQ files were removed using Clumpify (from BBTools suite of programs) from Department of Energy (Joint Genome Institute) with default parameters except dedupe = t (i.e., remove duplicates). Link: https://jgi.doe.gov/data-and-tools/bbtools/. The reads after duplicate removal were aligned to custom DSP genome from C57BL/6 (mm9) using Bowtie2 aligner (using very-sensitive-local option). Reads which had a minimum 100 base mapped length were used for counting reads to the specified regions using SAMtools. The regions used for counting were DSP2pt9_7-84, DSP2pt2_4698-4790, DSP2ptX1_9378-9457, DSP2ptX2_14022-14115, DSP2pt3_18693-18764, DSP2pt5_24559-24663.

### Chromosome conformation capture assay (3C)

3C analysis was performed as described (22). Briefly, 10^7^ CD19^+^ pro-B cells and CD4^+^CD8^+^ thymocytes derived from TCRβ × Rag2^−/−^ were used for 3C analysis. After digestion of crosslinked chromatin with Hind III, the ligated DNA was purified, and interaction efficiency measured by Taqman quantitative PCR method. Interactions in different 3C samples were normalized to α-amylase gene as described (22). The relative interaction efficiency between the anchor primer and target primer was calculated as Xspecific primer = 2Ct (a-amylase – specific primer). For primer efficiency calculation, equal moles of bacterial artificial chromosomes (BAC:RP23-351J19, RP23-269D13, RP23-363G23, and RP23-6A14) were mixed, digested with Hind III, re-ligated, and measured by Taqman quantitative PCR.

### Fluorescence *in situ* hybridization (FISH)

Pro-B cells from Rag2^−/−^ mice and DP thymocytes derived from TCRβ × Rag2^−/−^ mice were used for FISH analyses. FISH was performed as described by Guo et al. (22). Probes were as follows: named as RI (115051557-115227487) (BAC 373N4), RII (115944024-116124641) (BAC 70F21), and RIII (116777388-117011222) (BAC 368C22) (all the BACs were purchased from Thermofisher Scientific). Other position-specific 4–10 kb probes were generated by PCR using genomic DNA with the primers listed in Table S1. After probe hybridization to fixed cells (22), image acquisition was carried out using a Nikon T200 epifluorescence microscope. Twenty-five to thirty optical sections spaced by 0.2–0.3 μm acquired and the dataset was deconvoluted using NIS-Element software (Nikon). Statistical analysis of spatial distances between probes were done measured as previously described (25).

## Results

### Analysis of chromatin accessibility at *IgH* locus in DP thymocytes

**Figure 1:**
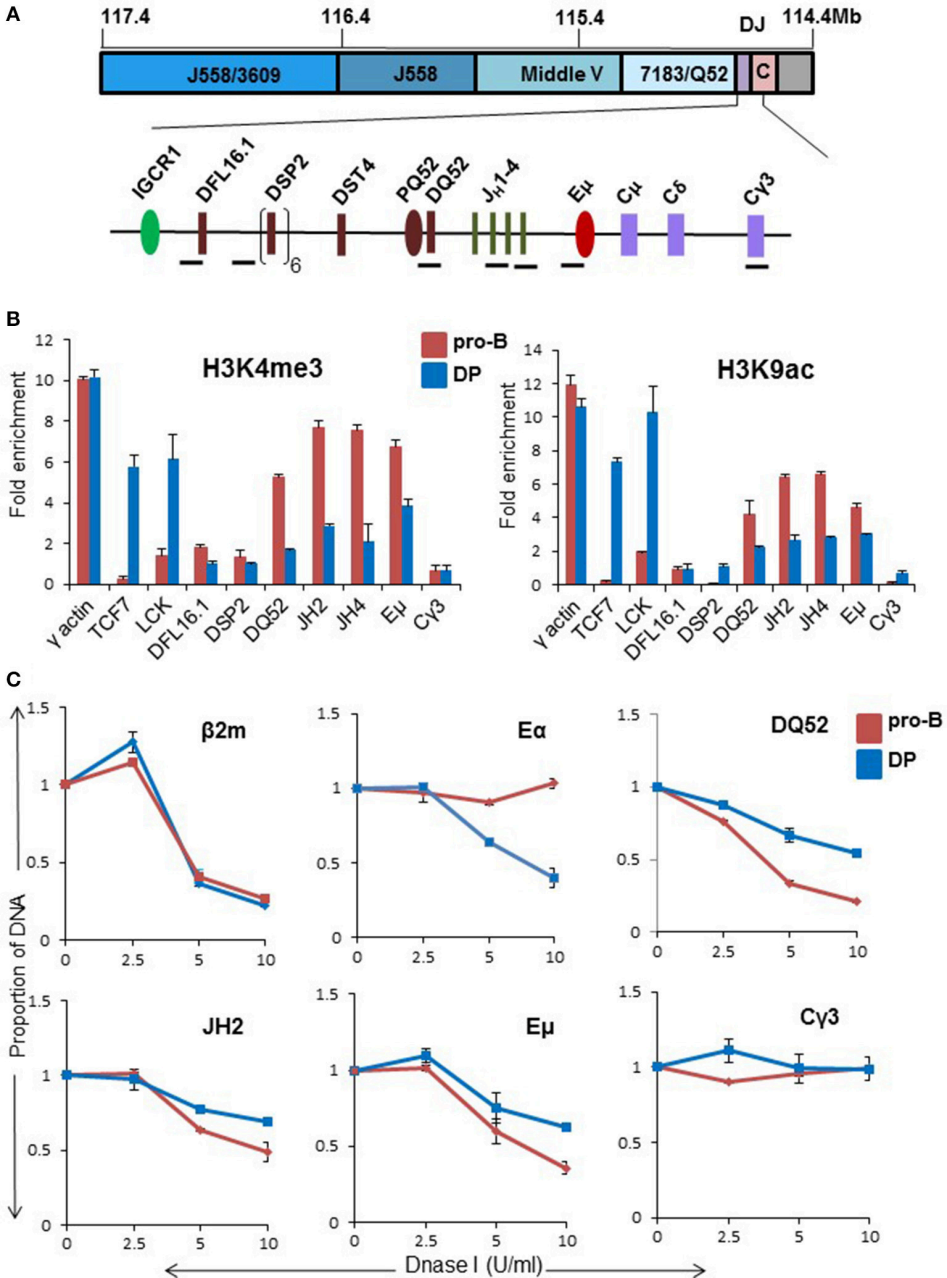
Chromatin state of the D_H_-C_H_ domain of the *IgH* locus in CD4^+^CD8^+^ DP thymocytes. **(A)** Top panel shows scale representation of the murine *IgH* locus based on mm9 (mouse reference genome). V_H_ gene families are indicated in blue. Purple box labeled as DJ contains all D_H_ and J_H_ gene segments, and pink box labeled as C contains exons of all *IgH* isotypes. The D_H_-C_H_ domain (120 kb) is expanded below to show the locations of ChIP amplicons (black bars). The intergenic control region 1 (IGCR1), DQ52 promoter (PQ52), and intronic enhancer (Eμ) are regulatory sequences that are marked by DNase I hypersensitive sites in pro-B cells. **(B)** CD19^+^ pro-B cells from bone marrow of Rag2^−/−^ and CD4^+^CD8^+^ thymocytes from TCRβ × Rag2^−/−^ transgenic mice were used in chromatin immunoprecipitation (ChIP) experiments using anti-H3K4me3 and H3K9ac antibodies. *TCF7* and *Lck* gene promoters served as positive controls in DP thymocytes. Cγ3 was used as a negative control and γ-actin served as a positive control in both cell types. For each independent experiment PCR was done in triplicate. Results shown are the mean of two independent experiments. Error bars represent standard error of the mean (*n* = 2). Y-axis shows enrichment of respective amplicons in the immunoprecipitate compared to an equal amount of input DNA as described in the methods. **(C)** DNase I sensitivity analysis of proximal part of *IgH* locus in pro-B cells and DP thymocytes. 10^7^ nuclei from CD19^+^ pro-B cells from Rag2^−/−^ mice and DP thymocytes from TCRβ × Rag2^−/−^ mice were treated with increasing concentration of DNase I (X-axis) followed by purification of genomic DNA. Equal amounts of DNA were used for amplification with the indicated primers. The signal from each amplicon was normalized to that from a β-globin amplicon for each DNase I concentration. β2m promoter served as a positive control while Cγ3 served as negative control. TCRα enhancer was used as an additional positive control for DP thymocytes. The data represents the mean of two independent experiments. Error bars represent standard error of the mean (*n* = 2).

*IgH* rearrangements can be seen early in lymphopoiesis in the common lymphoid precursor (CLP) and early thymic progenitors (ETP) in WT mice (5). Additionally, ongoing *IgH* rearrangements have been noted in CD4^−^CD8^−^ double negative (DN) and CD4^+^CD8^+^ double positive (DP) thymocytes (6). Because thymic rearrangements are Eμ-dependent, we tested the hypothesis that inadequate Eμ activation underlies partial *IgH* recombination in DP thymocytes. For this, we assayed several parameters of Eμ function in these cells. To maintain the *IgH* locus in germline configuration, we used DP thymocytes obtained from recombinase (Rag2)-deficient mice that expressed a transgenic TCRβ chain gene (TCRβ × Rag2^−/−^) (26, 27). We previously showed that Eμ regulates both H3K4me3 and H3K9ac histone modifications (associated with active chromatin) in the D_H_-Cμ region in primary pro-B cells (28). Both these marks were reduced in DP thymocytes compared to pro-B cells. *Tcf7* and *Lck* gene promoters served as T lineage-specific positive controls (Figures 1A,B). γ-actin promoter and Cγ3 amplicons served as positive and negative controls, respectively. These results were similar to reduced activation-specific histone modifications on Eμ-deficient alleles in pro-B cells (28). H3K27me3, a mark associated with inactive chromatin (29–31), did not differ substantially between pro-B cells and DP thymocytes (Figure S1A). We verified these observations with ChIP analysis of activation-specific mark (H3K4me3) in WT DP thymocytes (Figure S1B). Genome wide ChIP-seq studies (32, 33) of WT DP thymocytes also revealed activation modifications in the D_H_-Cμ part of the locus, however, the relative levels compared to pro-B cells could not be inferred from those studies. We conclude that the D_H_-Cμ part of the locus is partially active in DP cells.

Partial locus accessibility of the D_H_-Cμ domain in DP cells was further confirmed by DNase I sensitivity analysis. Nuclei from DP thymocytes or Rag2-deficient primary pro-B cells were treated with varying concentration of DNase I, followed by quantitative PCR to query specific regions. Signals from test amplicons were normalized to a beta-globin amplicon (24). We found reduced DNase I sensitivity in the region extending from DQ52 till Eμ in DP cells compared to primary pro-B cells (Figure 1C). Cγ3 and TCRα enhancer (Eα) served as negative and positive controls, respectively (Figure 1C). Overall, ChIP and accessibility assays revealed a partially active D_H_-Cμ domain in DP thymocytes, reminiscent of the state of Eμ-deleted *IgH* alleles in pro-B cells.

### Assessment of *IgH* intronic enhancer activity in DP thymocytes

**Figure 2:**
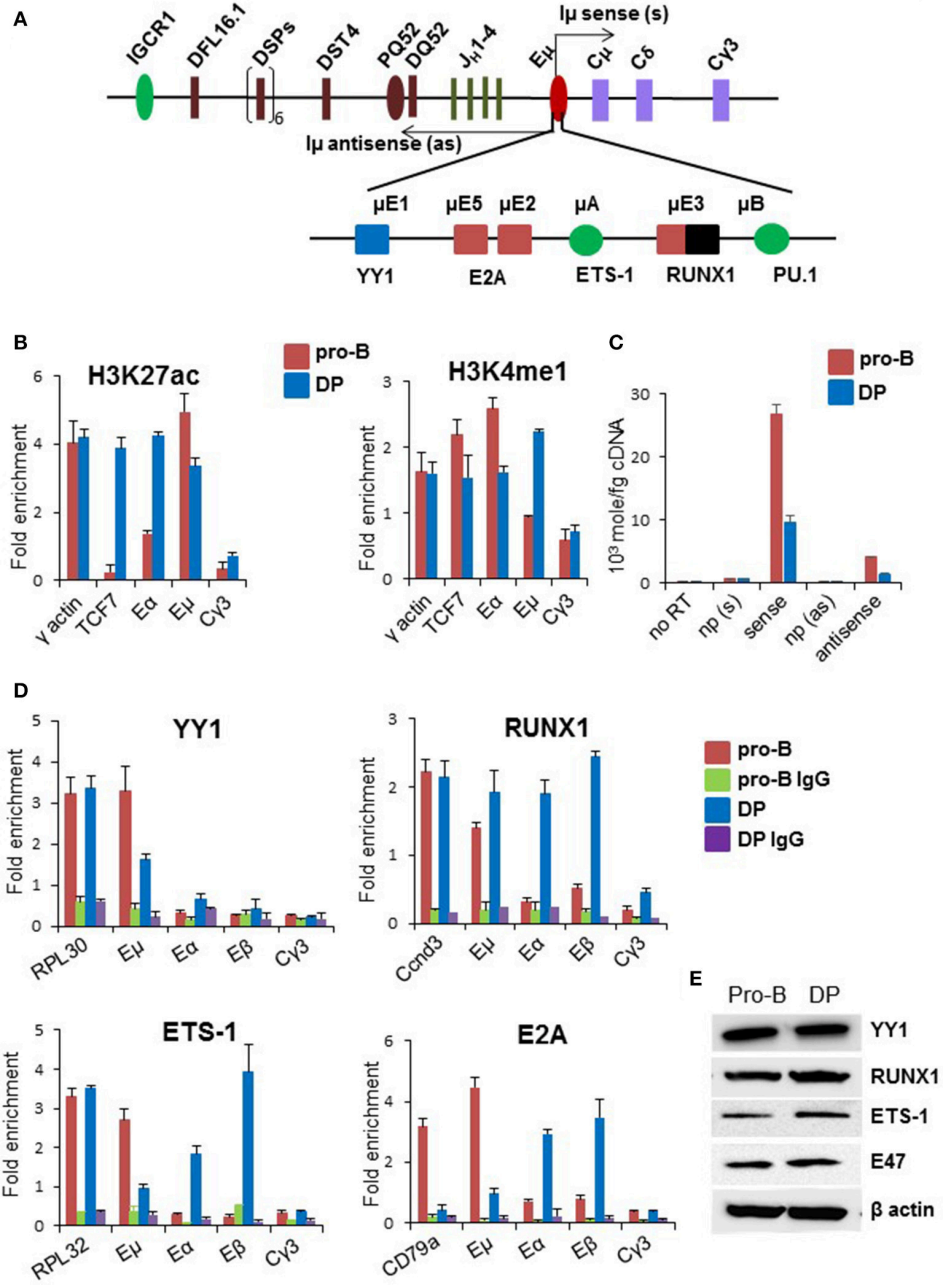
Status of Eμ enhancer in DP thymocytes. **(A)** Enhancer elements and transcription factors that bind to these sites are shown below the schematic of the D_H_-C_H_ part of the *IgH* locus. Arrows originating from the enhancer represent bi-directionally transcribed eRNAs named as Iμ sense and Iμ antisense. **(B)** Enhancer-associated histone modifications were scored by ChIP using anti-H3K4me1 and anti-H3K27ac antibodies in CD19^+^ pro-B cells and DP thymocytes derived from TCRβ × Rag2^−/−^ transgenic mice. Y-axis represents fold enrichment of the indicated amplicon in the immunoprecipitate compared to an equal amount of input DNA. For each independent experiment PCR was done in triplicate. The data shown is the mean of two independent ChIP experiments. Error bars represent standard error of the mean (*n* = 2). γ-actin was used as a positive control, *TCF7* enhancer was used as a positive control for T-lineage-specific gene and Cγ3 was used as a negative control. **(C)** Levels of eRNAs that originate within the enhancer in DP thymocytes and pro-B cells. Reverse transcription was carried out with strand specific primers, or no primer (np), followed by amplification with primers that score for sense and antisense Iμ transcripts. RNA amounts were calculated based on a standard curve obtained from serial dilutions of 1 kb DNA spanning both sense and antisense transcribed region. **(D)** Transcription factor binding to Eμ was assayed by chromatin immunoprecipitation using antibodies directed against the indicated factors. Enrichment of specific amplicons in co-precipitated DNA was calculated relative to an equal amount of input DNA (Y-axis). Error bars represent standard error of the mean (*n* = 2). Positive controls for each transcription factor correspond to the first set of bars in each graph. IgG served as additional negative control. **(E)** YY1, E2A, Ets-1, and RUNX1 expression in DP thymocytes and Rag2^−/−^ pro-B cells were assayed by immunoblotting with the respective antibodies.

We directly examined the state of Eμ with additional ChIP and transcription studies. Based on genome wide studies, the prevailing view is that poised enhancers are marked by H3K4me1 and H3K27me3, whereas active enhancers are marked by H3K27ac (34–36). Consistent with this view, Eμ sequences were enriched for H3K27ac, with close to basal level of H3K4me1 in pro-B cells (Figure 2B). By contrast, Eμ was marked by both H3K27ac as well as H3K4me1 in DP thymocytes. Eα and TCF7 enhancers, which served as positive controls in DP thymocytes, had high levels of H3K27ac in DP cells but not in pro-B cells. Increased H3K4me1 at Eμ in DP cells compared to pro-B cells was consistent with its being partially active in DP cells. This conclusion was substantiated by reduced Eμ-associated eRNA levels in DP cells (Figure 2C).

Eμ binds several transcription factors (37–41) (Figure 2A). To understand the basis for partial Eμ activity, we compared transcription factor occupancy at the enhancer in DP (TCRβ × Rag2^−/−^) and pro-B cells by ChIP. We found that YY1 levels at Eμ were reduced in DP cells compared to pro-B cells, RUNX1 binding to Eμ was comparable in pro-B and DP cells, whereas E2A and Ets-1 binding was substantially lower (Figure 2D). We assayed several of these factors in DP thymocytes enriched from WT C57BL/6 mice and observed similar trends. YY1 and RUNX1 bound Eμ in WT DP cells, whereas E2A binding was lower at Eμ compared to Eβ and Eα (Figures S2A–E). The observed changes were not due to differential expression of these transcription factors (Figure 2E). To determine whether absence of E2A from Eμ could be accounted for by a different basic helix-loop-helix protein, we carried out ChIP with anti-HEB antibodies in WT DP cells. HEB binding to Eμ was easily evident in these cells (Figure S2E). We propose that partial and perhaps inappropriate transcription factor binding to Eμ underlies its compromised activity in DP cells.

### D_H_ to J_H_ recombination in thymocytes

**Figure 3:**
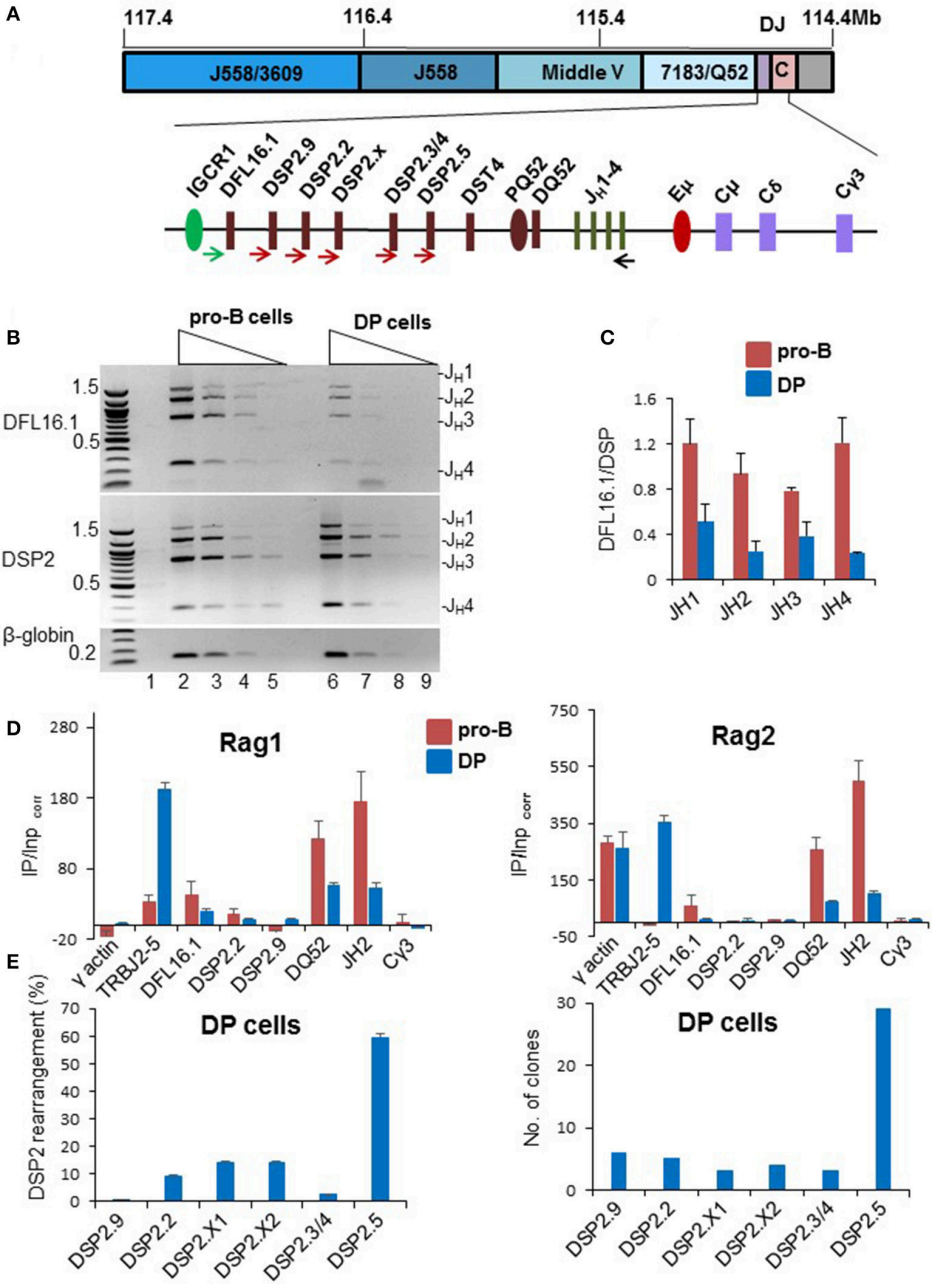
Analysis of D_H_ rearrangements in pro-B cells and DP thymocytes. **(A)** Schematic of D_H_ gene segments and positions of primers used to amplify DJ_H_ recombinations. **(B)** Genomic DNA prepared from C57BL/6 pro-B cells and DP thymocytes (Figures S3B–D) were used in PCR reactions with a 5′ primer located upstream of DFL16.1 (green arrow), or one that hybridizes to all 6 DSP2 gene segments (brown arrows), and a 3′ primer located after J_H_4 (black arrow). Four-fold serially diluted DNAs were used for PCR amplification reaction followed by separation of the products by electrophoresis through 1% agarose gels. PCR analysis was carried out with two independent preparations of pro-B and DP cells and the data shown is one representative example. An amplicon from the β-globin gene was used as loading control and a no-DNA control is shown in lane 1. **(C)** DSP2 and DFL16.1 utilization in pro-B cells and DP thymocytes was calculated after band intensity quantitations from two different gels using Gene Tool. Error bars represent standard error of the mean between two independent gel quantitations. **(D)** Rag1 and Rag2 binding to the *IgH* locus was evaluated by chromatin immunoprecipitation. Immunoprecipitated genomic DNA and input DNA were used for qPCR and fold enrichment was calculated as described by Ji et al. (23). DP thymocytes were compared to the D345 pro-B cell line as indicated. γ-actin served as positive control for Rag2 but negative control for Rag1. Cγ3 is used as negative control for both Rag1 and Rag2. TCRJ_β_2 gene (TRBJ2-5) is used as additional positive control for DP thymocytes. For each independent experiment PCR was done in triplicate. Data shown is the mean of two independent experiments. Error bars represent standard error of mean (*n* = 2). **(E)** Utilization of DSP2 gene segments in DJ_H_ junctions in DP thymocytes from C57BL/6 mice. DSP2-J_H_1 recombined products were amplified using a forward pan-DSP2 primer and a reverse primer located 3′ of J_H_1. Amplification products were gel purified followed by adapter ligation and sequencing (left panel). The number of reads aligned to each DSP2 gene are shown in Figure S3F. Percentage of reads mapping to indicated DSP2 gene segments are shown (after removal of redundant reads). DSP2-J_H_1 amplification products were also cloned into pGEM-T vector and 60 clones were sequenced (right panel). Number of clones with each gene segment are shown in the bar graph. Thirty clones were sequenced each time from two different PCR amplification. 50 out of 60 clones that had unique junctional sequences are represented in the bar graph.

During D_H_ recombination in pro-B cells the 5′-most and 3′-most D_H_ gene segments, DFL16.1 and DQ52, are used most frequently (42–44). Even amongst intervening DSP2 gene segments, those located at either ends of the cluster, rearrange more frequently (Figure S3A). This distribution has been proposed to be due to the looped configuration of *IgH* alleles that places DFL16.1 closest to the J_H_-associated recombination center (22, 45). Though DJ_H_ junctions were noted in DP cells many years ago, D_H_ utilization in thymocytes has not been quantified. Because patterns of D_H_ utilization may provide mechanistic insights, we assayed D_H_ recombination frequency in DP cells. We used 5′ primers specific for DFL16.1, or a pan DSP2 primer that binds to six DSP2 gene segments, with a 3′ primer located 3′ of J_H_4 (Figure 3A) to amplify DJ_H_ rearrangements using genomic DNA from bone marrow pro-B cells or DP thymocytes (Figures S3B–D). Following fractionation by agarose gel electrophoresis and quantitation, we observed the expected over-utilization of DFL16.1 in primary pro-B cells (Figure 3B, lanes 2–5). The equivalent intensity of DFL16.1 and DSP2 rearrangements in the gel image results from the pan DSP2 primer scoring for 6 different DSP2 gene segments. In contrast, the proportion of DFL16.1 usage was greatly reduced in DP thymocytes (Figure 3B, lanes 6–9), quantitated in Figure 3C. We also observed low occupancy of recombinase proteins at *IgH* locus in DP thymocytes relative to pro-B cells (Figure 3D). Reduced utilization of DFL16.1 in DP thymocytes suggests that the configuration of the *IgH* locus within which D_H_ rearrangements occur in DP cells differs from that in pro-B cells.

In pro-B cells increased utilization of DFL16.1 has been attributed to spatial proximity of DFL16.1 to the J_H_-associated recombination center (RC) via Eμ/IGCR1 interaction. Our analysis of D_H_ utilization in DP cells suggested that Eμ may not efficiently recruit DFL16.1 to the recombination center in these cells. To test the possibility, we carried out chromosome conformation capture (3C) assay in Rag2^−/−^ pro-B cells and DP thymocytes derived from TCRβ × Rag2^−/−^ transgenic mice. Eμ interactions with IGCR1 and with HS5, were reduced in DP thymocytes compared to pro-B cells (Figure S3E). We used Eα-TEAp interaction as a positive control in thymocytes and Eμ-HS5 served as positive control for pro-B cells. Eμ-RPL32 was used as an out of locus negative control. These data indicated that reduced DFL16.1 utilization in DP thymocytes may be the consequence of weaker association between Eμ and IGCR1.

To determine whether skewed D_H_ usage was also evident amongst the closely related DSP2 gene segments, we amplified DSP2-J_H_1 rearrangements from DP cell DNA and sequenced the resulting amplicons. We identified individual DSP2 gene segments from 5' flanking sequences and non-redundant sequence reads were identified as described in the Materials and Methods section. Representative sequences from DP thymocytes confirmed unique DJ_H_ rearrangement-associated junctional diversity (Figure S3F and Table S2). Quantitation of DSP2 utilization revealed striking over-utilization of the 3′-most DSP2 gene segment DSP2.5 in these rearrangements (Figure 3E left panel). To further confirm sequencing results, we cloned and sequenced DSP2-J_H_1 junctions from DP cells. Thirty out of 50 unique cloned sequences contained DSP2.5 (Figure 3E right panel). Sequences of recombined products from colony sequencing are shown in Table S3. We conclude that the D_H_ rearrangements in DP thymocytes preferentially utilize gene segments located near the 3′ part of the D_H_ locus.

### Basis for lack of V_H_ recombination in thymocytes

V_H_ recombination does not occur in DP thymocytes on WT *IgH* alleles, despite availability of a DJ_H_ junction in recombinase-expressing DP cells. One possibility is that these cells do not survive long enough to undergo two sequential recombination events. Alternatively, V_H_ gene recombination may be prohibited for mechanistic reasons. An essential requirement for V_H_ recombination is for these gene segments come into proximity of DJ_H_ junction to permit Rag1/2-dependent synapsis. Current evidence indicates that distal and proximal V_H_ genes are differently regulated in pro-B cells. We previously proposed that distal V_H_ gene segments come close to the 3′ *IgH* domain via three inter-dependent steps regulated by three different transcription factors (25). The first step uses CTCF to fold the V_H_ region into domains of several hundred kb. The second step further compacts the distal V_H_ region using Pax5 and the third step brings the pre-folded V_H_ region close to the DJ_H_ part of the locus by a process that requires YY1. By contrast, Pax5 and YY1 are not required for proximal V_H_ rearrangements (7, 46), suggesting that CTCF-dependent interactions are sufficient.

**Figure 4:**
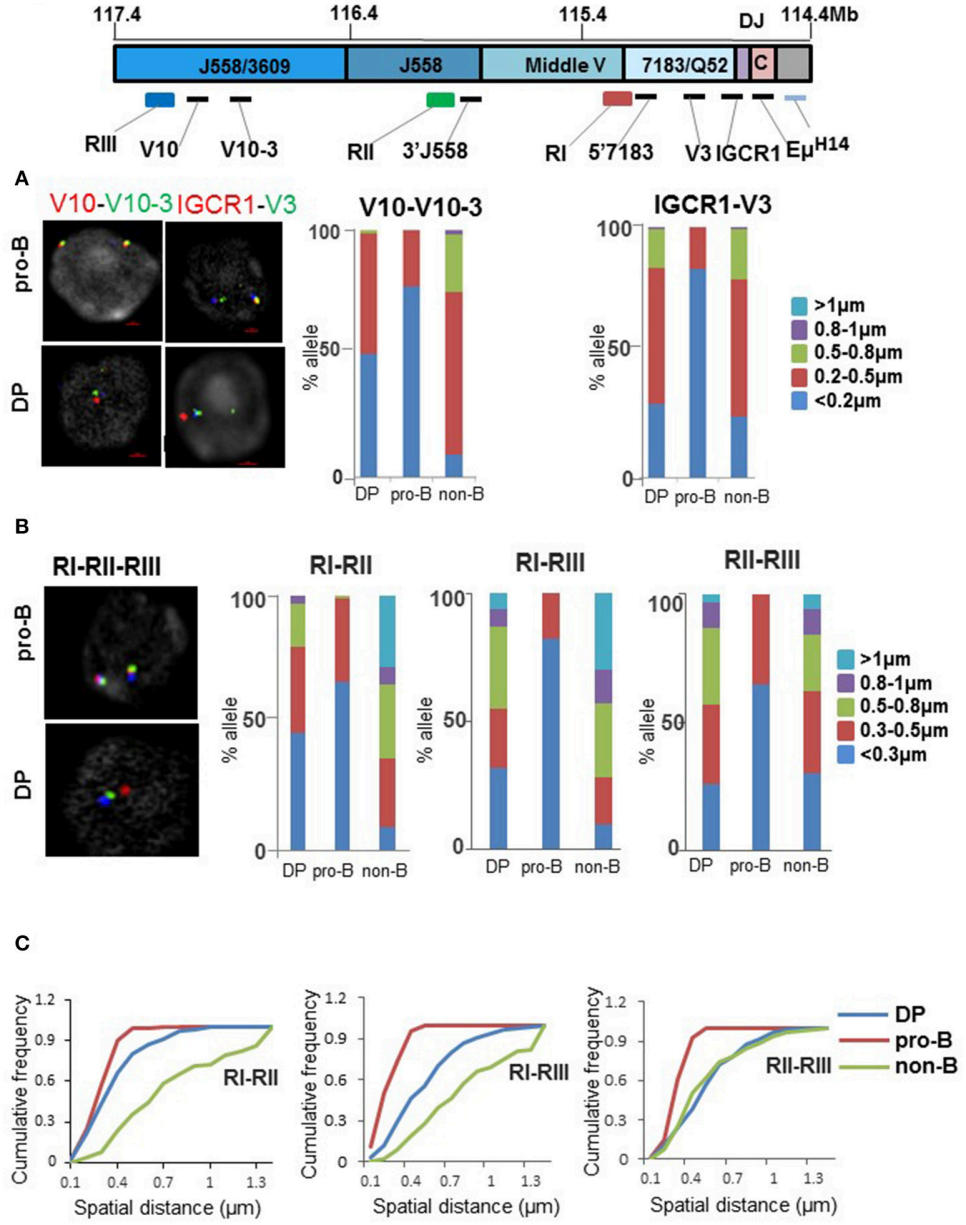
Status of V_H_ locus in pro-B and DP thymocytes. **(A)** Fluorescence *in situ* hybridization (FISH) analyses of *IgH* alleles in pro-B cells and DP thymocytes. Top panel shows the unrearranged *IgH* locus with locations of FISH probes. Probes labeled as V10, V10-3, V3, and IGCR1 were generated by PCR amplification from bacterial artificial chromosome (BAC) DNA and range in length from 4 to 10 kb. RI-RIII are BACs. Two color DNA-FISH were carried out for CTCF-dependent interactions using FISH probes indicated on the top. *IgH* alleles were marked with BAC RP23-201H14 (light blue) which is located 200 kb 3′ of HS4. Representative nuclei are shown. Spatial distances between probes were measured as described in (22). Bar graph shows percentage of *IgH* alleles in which the distance between probes fell in the ranges shown in different colors (*n* = 100). **(B)** BAC probes RI, RII, and RIII labeled as indicated, were hybridized simultaneously to pro-B cells, DP cells, and non-B cells derived from bone marrow of Rag2^−/−^ mice. The percentage of *IgH* alleles in which distances between BAC probes fell in the indicated ranges are shown in the bar graph (*n* = 100). **(C)** Cumulative frequency distribution of spatial distances for each probe combination RI-RII, RI-RIII, and RII-RIII are indicated.

We used fluorescence *in situ* hybridization (FISH) to investigate the conformational state of the *IgH* locus in DP thymocytes. The first level of (CTCF-dependent) compaction was analyzed with probes located in different parts of the V_H_ locus. We found that the probe pair V10-V10-3, located in the distal V_H_ region, were comparably positioned in pro-B and DP cells but different in non-B lineage bone marrow cells (Figure 4A). By contrast spatial proximity of IGCR1-V3 probe pairs, querying proximal V_H_ interactions, was similar in DP and non-B cells (Figure 4A and Figure S4A). This was neither due to differential expression of CTCF between DP cells and pro-B cells (data not shown) nor due to lack of CTCF binding to the *IgH* locus in these cell types based either on genome-wide assays or ChIP followed by quantitative PCR (Figures S4B,C). We conclude that presence or absence of CTCF-dependent interactions in DP cells depends on their location within the *IgH* locus. Since 60–70% of CTCF sites are also bound by RAD21 in *IgH* locus, we assayed RAD21 binding at *IgH* locus. We found that RAD21 binding was reduced at proximal V_H_ gene segments (Figure S4C), consistent with recent observation of Loguercio et al. (47). We used additional primers from the distal V_H_ region to assay RAD21 binding in WT DP thymocytes and Rag2^−/−^ pro-B cells (Figure S4D). RAD21 binding varied between the 3 new amplicons, precluding any definitive conclusions regarding RAD21 recruitment to CTCF-bound sites in the distal V_H_J558 region.

In our working model a CTCF looped distal V_H_ region is further compacted by Pax5. Because Pax5 is not expressed in the T lineage, it was likely that this second level of compaction would be absent in DP cells. We verified this prediction using bacterial artificial chromosome (BAC) probes RII and RIII that span the distal V_H_ region (Figure 4B). The proximal 1 Mb of the V_H_ region was examined using BAC probes RI and RII. This association was more similar in DP and pro-B cells than in non-B cells (Figure 4B and quantitated in Figure 4C). The final step of locus compaction brings a pre-folded V_H_ region into proximity of the D_H_ -Cμ domain mediated by the transcription factor YY1. We queried this interaction with probes located near Eμ and different parts of the locus. Eμ interaction with both proximal (V_H_7183) and distal (V_H_J558) parts of the locus was substantially disrupted in DP thymocytes (Figures S4E,F). Overall, V_H_ region compaction is affected at multiple levels in DP cells compared to pro-B cells.

## Discussion

Antigen receptor genes recombine in a lineage- and stage-specific manner using enhancers to regulate chromatin structure and, thereby, locus accessibility to recombinase. One exception to this rule is that up to 50% of T lymphocytes contain partially rearranged *IgH* alleles, demonstrating that *IgH* rearrangements are not limited to B lymphocytes (2). These rearrangements likely occur at multiple developmental stages including ETP, DN and DP stages (5). Here we probed *IgH* locus structure in DP thymocytes to address three questions. First, why do D_H_ gene segments recombine in DP cells, and does this step follow the same rules as in pro-B cells? Second, why does V_H_ recombination not occur in in DP cells? And third, do these properties provide insights into mechanisms that regulate tissue-specific locus activation?

### Regulation of D_H_ recombination in thymocytes

Earlier observations that Eμ deficiency abrogates D_H_ recombination in thymocytes provides a preliminary answer to the first question (21). Namely, D_H_ gene segments rearrange because Eμ makes the *IgH* locus accessible to recombinase in DP cells. However, we now show that the mechanism by which Eμ is activated and consequences of Eμ activity differs between DP and pro-B cells. We demonstrate that the constellation of transcription factors that bind Eμ in DP cells differs considerably from those that activate it in pro-B cells. Significantly missing from the enhancer in DP cells are the ETS-domain proteins Ets-1 and the bHLH protein E2A. The latter may be substituted by the related protein HEB, and we did not check for PU.1 binding to Eμ because this protein is not expressed in DP cells (48). Additionally, YY1 binding to Eμ is reduced in DP cells. Thus, the enhancer milieu in DP thymocytes is quite different from that in pro-B cells. We suggest that this configuration results in inappropriate enhancer activity, leading to differences in epigenetic features, chromosome conformation, production of eRNAs and RAG recruitment to the *IgH* locus in DP cells compared to pro-B cells.

The ensemble average nature of ChIP studies precludes a molecular definition of “inappropriate enhancer activity.” The most clear difference between pro-B cells and DP thymocytes in regard to Eμ occupancy by transcription factors are the absence of both Ets-1 or PU.1 and the substitution of E2A by HEB at Eμ in DP cells. The current analysis does not rule out that Ets-1 and PU.1 may also be replaced by another ETS-domain protein in thymocytes. These features appear to characterize most DP thymocytes, by contrast, YY1 binding in DP cells is reduced to ~50% the level in pro-B cells. It is impossible to distinguish whether the YY1 bound state represents 50% cells or 50% of alleles. Adding up these features leads to a view of Eμ bound by RUNX1, HEB, and YY1 on a subset of alleles in DP thymocytes. Our working hypothesis is that absence of the right combination of ETS-domain proteins at the Eμ effectively cripples optimal enhancer activity. This is reflected in maintenance of H3K4me1, a mark of poised enhancers, at Eμ in DP thymocytes.

A functional consequence of inappropriate Eμ activity is the markedly different utilization of D_H_ gene segments in DP cells compared to pro-B cells. DFL16.1, the 5′-most D_H_ gene segment, no longer dominates DJ_H_ junctions, and DSP2 rearrangements are skewed toward the 3′-most DSP2.5 gene segment in DP cells. Reduced DFL16.1 rearrangements can be understood in part by reduced Eμ/IGCR1 interactions in DP cells that, in pro-B cells, bring this gene segment into spatial proximity of the recombination center. Skewed utilization of DSP2.5 is harder to explain. In pro-B cells DSP2 gene segments at either ends of the cluster rearrange more frequently than those that lie in the middle (42–44). That is, DSP2.2 and 2.9 (at the 5′ end) and DSP2.5 (at the 3′ end) recombine more than DSP2.X and 2.3 (in the middle). We have previously proposed that this pattern arises from repeat-induced gene silencing (RIGS), a form of RNA-interference initiated heterochromatin formation, of the DSP2 repeat region (49). Our working model is that extensive use of DSP2.5 in thymocytes reflects reduced DSP2.2 and 2.9 utilization (rather than specific activation of DSP2.5) due to weakened RIGS as well movement of these gene segments away from the RC due to reduced Eμ/IGCR1 interaction. RAG proteins tracking (50) from the relatively poor *IgH* RC in DP cells would thus encounter the DSP2.5 RSS first to initiate rearrangements. Accordingly, most DSP2.5 rearrangements occur by deletion rather than inversion (data not shown).

### Lack of V_H_ recombination in thymocytes

Our studies reveal several mechanisms that contribute to the absence of V_H_ rearrangements in DP thymocytes. First, conformational compaction of the distal V_H_ region is disrupted in DP cells. This may be attributed to the absence of Pax5 expression in these cells, since proximity between BAC probes RII and RIII is similarly dislocated in Pax5-deficient pro-B cells (51). Second, CTCF-dependent interaction between IGCR1 and the proximal V_H_ genes does not occur in DP cells, though CTCF binding throughout the *IgH* locus is comparable to that in pro-B cells. We surmise that absence of these interactions is due to reduced cohesin recruitment to V_H_ region CTCF sites in DP cells. The disconnect between CTCF binding and cohesin binding at the *IgH* locus has been previously noted, though the molecular mechanism by which this occurs remains obscure (47, 52). Our observations identify chromatin structural consequences of this disconnect that provide a plausible explanation for the lack of proximal V_H_ recombination in DP cells. Third, an attenuated Eμ in DP cells may be unable to activate DJ_H_ junctions for secondary V_H_ recombination. One piece of evidence in support of this proposal is that DJ_H_ junctions in pro-B cells get hypo-methylated at CpG residues, whereas they remain hyper-methylated in DP cells and on Eμ -deficient *IgH* alleles in pro-B cells (53). We hypothesize that the cumulative effect of these processes inhibits V_H_ rearrangements in DP thymocytes. However, we cannot rule out the more mundane explanation that absence of V_H_ recombination is the stochastic consequence of reduced RAG1/2 recruitment by a sub-optimally active Eμ.

Our working hypothesis warrants consideration of situations in which V_H_ recombination can be induced in DP thymocytes. Two prominent circumstances have been documented. First, ectopic expression of Pax5 in DP cells results in proximal V_H_7183 gene rearrangements (6, 7). We surmise that this may be directed by Pax5 binding to sites within V_H_7183 genes as shown by Revilla et al. (54). Whether Pax5 expression also compacts distal V_H_ genes in DP cells remains to be determined. Second, disruption of IGCR1 leads to rearrangement of the 3′-most V_H_81X in DP cells (8). Based on the observation that Eμ loops to a CTCF binding site close to V_H_81X on IGCR1-mutated alleles to promote highly specific rearrangement of this gene segment in pro-B cells (55), we infer that a similar mechanism may also operate in DP thymocytes even with a sub-optimal Eμ.

### Tissue-specific enhancer activation

The state of Eμ in DP cells has implications for mechanisms of tissue-specific enhancer activation. One of the interesting features is that differences in Eμ occupancy by many factors, such as Ets-1, E2A, and YY1, occur despite comparable expression of these factors in pro-B and DP cells. These observations substantiate the idea that one or more key tissue-specific factor directs optimal enhancer occupancy. In the case of Eμ, such a function can be ascribed to PU.1 which may recruit or stabilize Ets-1 binding to the enhancer in pro-B cells; in its absence Ets-1 is not recruited to the enhancer in DP cells. Extending this line of reasoning suggests that the PU.1/Ets-1 combination is required for optimal YY1 and E47 binding. Alternatively, the ETS protein milieu of DP cells may exclude Ets-1 binding by mass action. That is, DP cells may contain other ETS proteins with higher affinity to sites in Eμ, or are present in greater abundance, that competitively displace Ets-1. Similar considerations may explain the substitution of E47 by HEB at Eμ in DP cells.

Taken together with earlier observations that Eμ-deleted *IgH* alleles bear certain hallmarks of locus activation in pro-B cells, our results lead to the following working hypothesis about tissue-specific Eμ function. Locus-specific changes that occur in lymphoid lineage cells permits transcription factor access to Eμ. The combination of factors present in pro-B cells result in optimal Eμ function. In the absence of the correct constellation of factors in the T lineage, Eμ is occupied by factors that are available in that milieu. However, the combination of inappropriate factor binding, and empty sites leads to sub-optimal function. Our observations demonstrate that enhancers can be partially (or inappropriately) occupied by transcription factors in the wrong cell type. Such occupancy could underlie their H3K4me1 marking in many different cell types, till the right set of factors bind to activate the enhancer, reduces H3K4me1 level and mark it with H3K27ac.

## Author contributions

GK performed all the ChIP, RNA, and DJ recombination experiments. TG and HD performed DNA-FISH. SD, WW, and KB carried out sequencing of recombined alleles, and bioinformatic analysis of DJ-seq data. GK prepared all the figures. GK and RS wrote the manuscript.

### Conflict of interest statement

The authors declare that the research was conducted in the absence of any commercial or financial relationships that could be construed as a potential conflict of interest.
