# Supplementary material for: Misregulation of the IgH Locus in Thymocytes
Source: Front Immunol. 2018 Nov 13;9:2426. doi: 10.3389/fimmu.2018.02426 (PMC6244664; doi:10.3389/fimmu.2018.02426)
Supplement: Supplementary file 1 [file Data_Sheet_1.pdf]

Figure S1

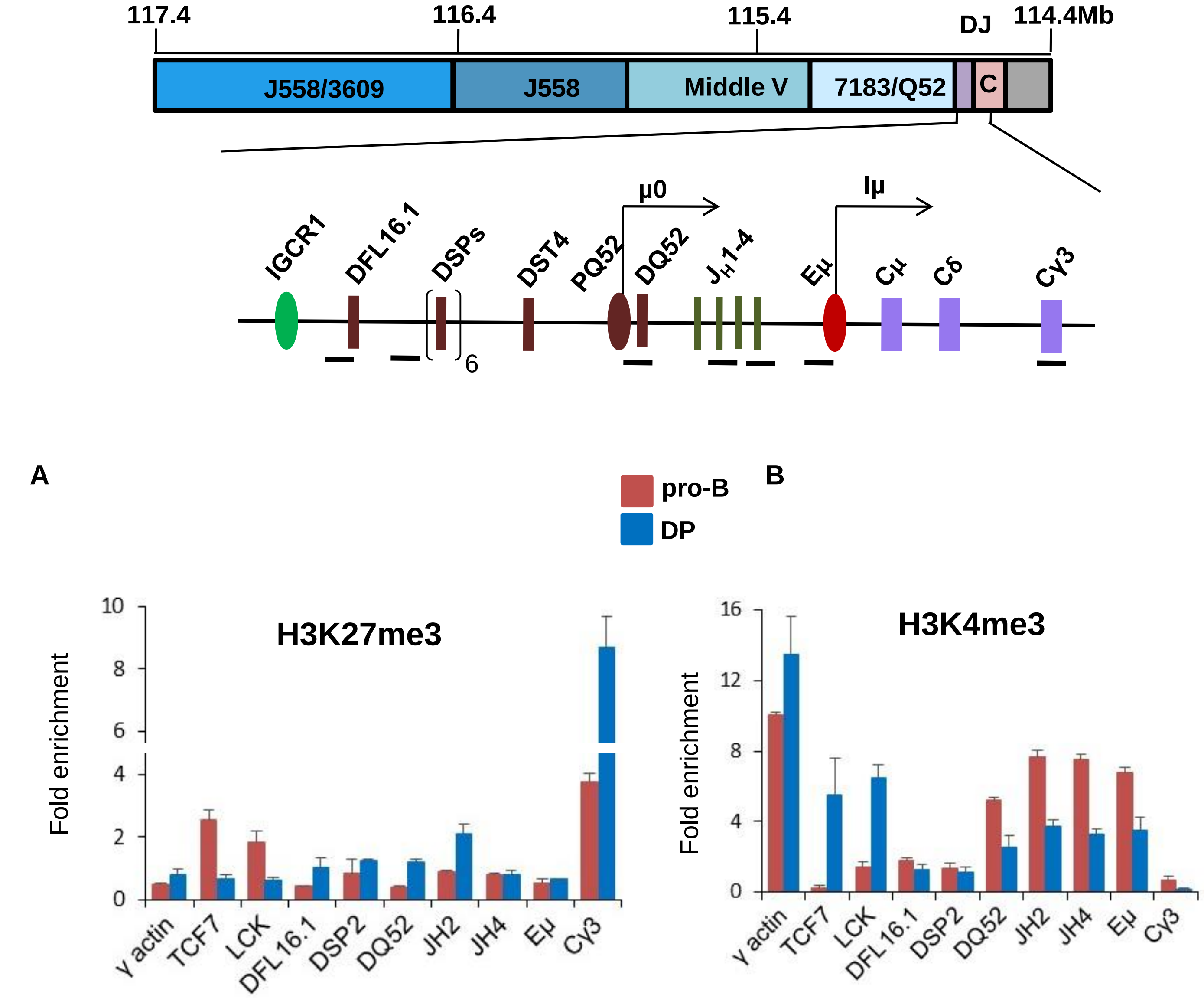
117.4
116.4
115.4
DJ
114.4Mb
J558/3609
J558
Middle V
7183/Q52
C
μ0
Iμ
IGCR1
DFL16.1
DSPs
DST4
PQ52
DQ52
J_H1-4
Eμ
Cμ
Cδ
Cγ3
6
A
B
pro-B
DP
H3K27me3
Fold enrichment
10
8
6
4
2
0
γ actin
TCF7
LCK
DFL16.1
DSP2
DQ52
JH2
JH4
Eμ
Cγ3
H3K4me3
Fold enrichment
16
12
8
4
0
γ actin
TCF7
LCK
DFL16.1
DSP2
DQ52
JH2
JH4
Eμ
Cγ3

Figure S2

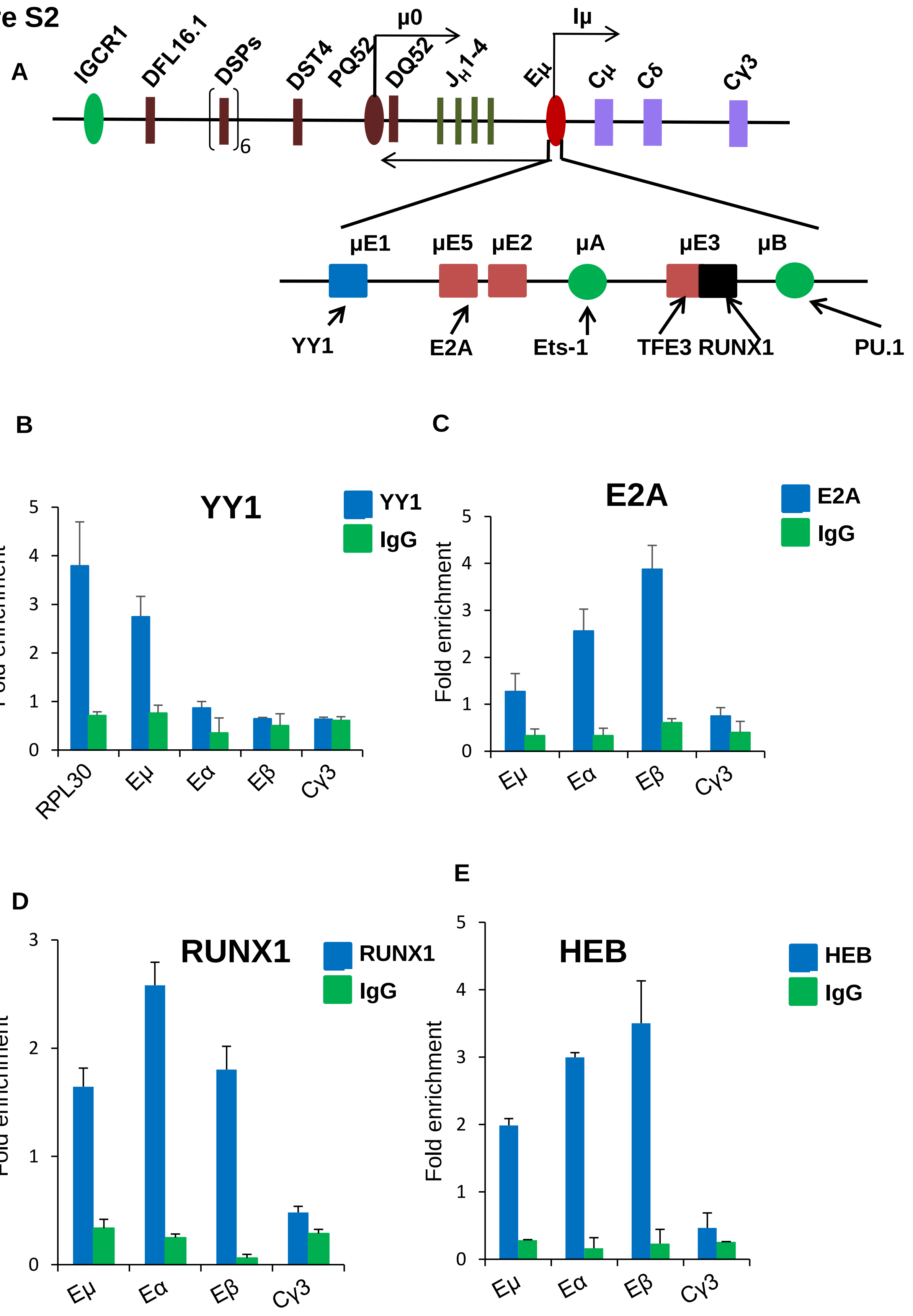
A
IGCR1
DFL16.1
DSPs
6
DST4
PQ52
DQ52
μ0
JH1-4
Eμ
Iμ
Cμ
Cδ
Cγ3
μE1
μE5
μE2
μA
μE3
μB
YY1
E2A
Ets-1
TFE3
RUNX1
PU.1
B
YY1
YY1
IgG
Fold enrichment
0
1
2
3
4
5
RPL30
Eμ
Eα
Eβ
Cγ3
C
E2A
E2A
IgG
Fold enrichment
0
1
2
3
4
5
Eμ
Eα
Eβ
Cγ3
D
RUNX1
RUNX1
IgG
Fold enrichment
0
1
2
3
Eμ
Eα
Eβ
Cγ3
E
HEB
HEB
IgG
Fold enrichment
0
1
2
3
4
5
Eμ
Eα
Eβ
Cγ3

Figure S3

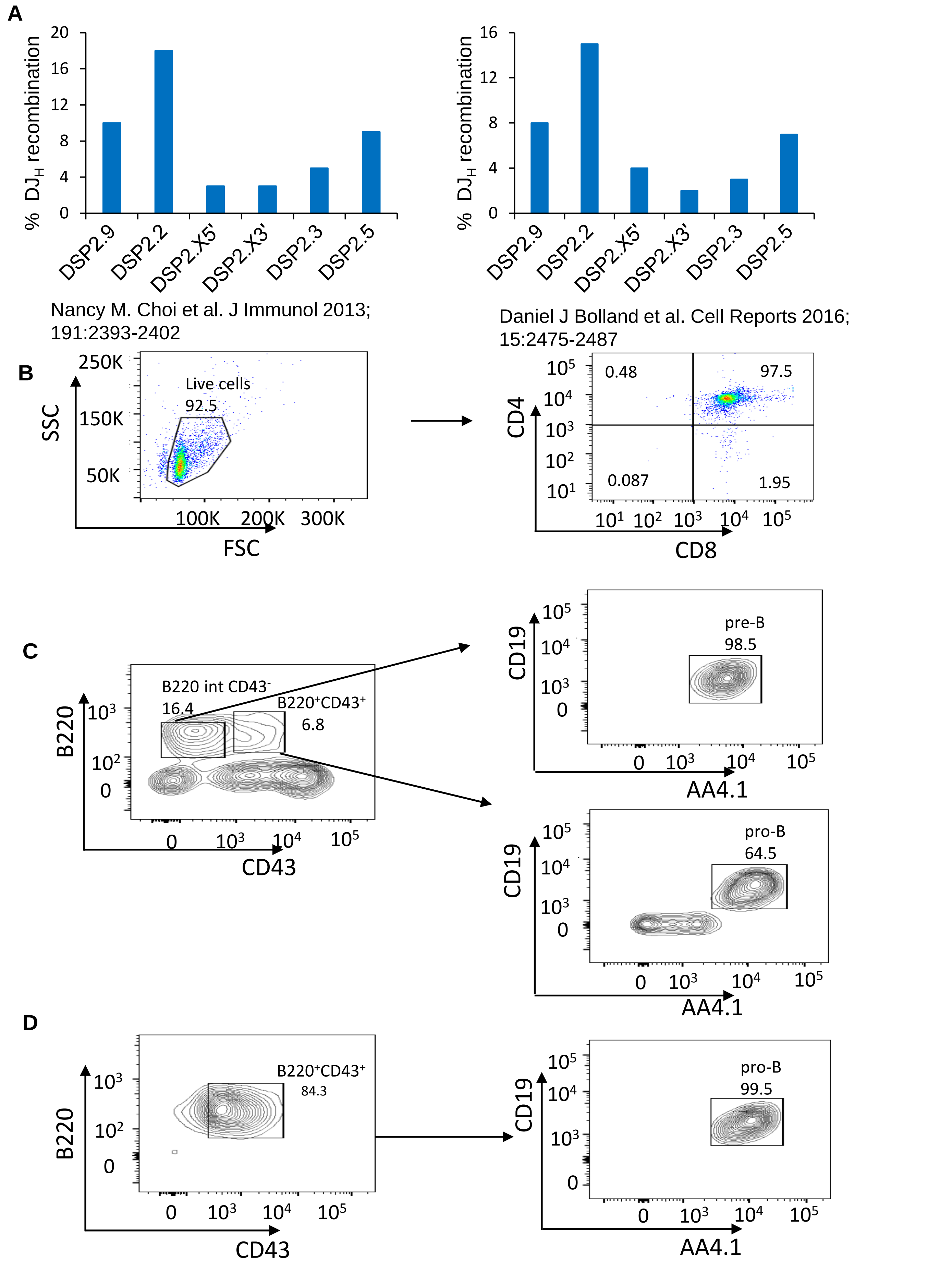

A
% DJH recombination
DSP2.9
DSP2.2
DSP2.X5'
DSP2.X3'
DSP2.3
DSP2.5
Nancy M. Choi et al. J Immunol 2013; 191:2393-2402
Daniel J Bolland et al. Cell Reports 2016; 15:2475-2487
B
SSC
FSC
Live cells
92.5
CD4
CD8
0.48
97.5
0.087
1.95
C
B220
CD43
B220 int CD43-
16.4
B220+CD43+
6.8
CD19
AA4.1
pre-B
98.5
pro-B
64.5
D
B220+CD43+
84.3
pro-B
99.5

Figure S3

E

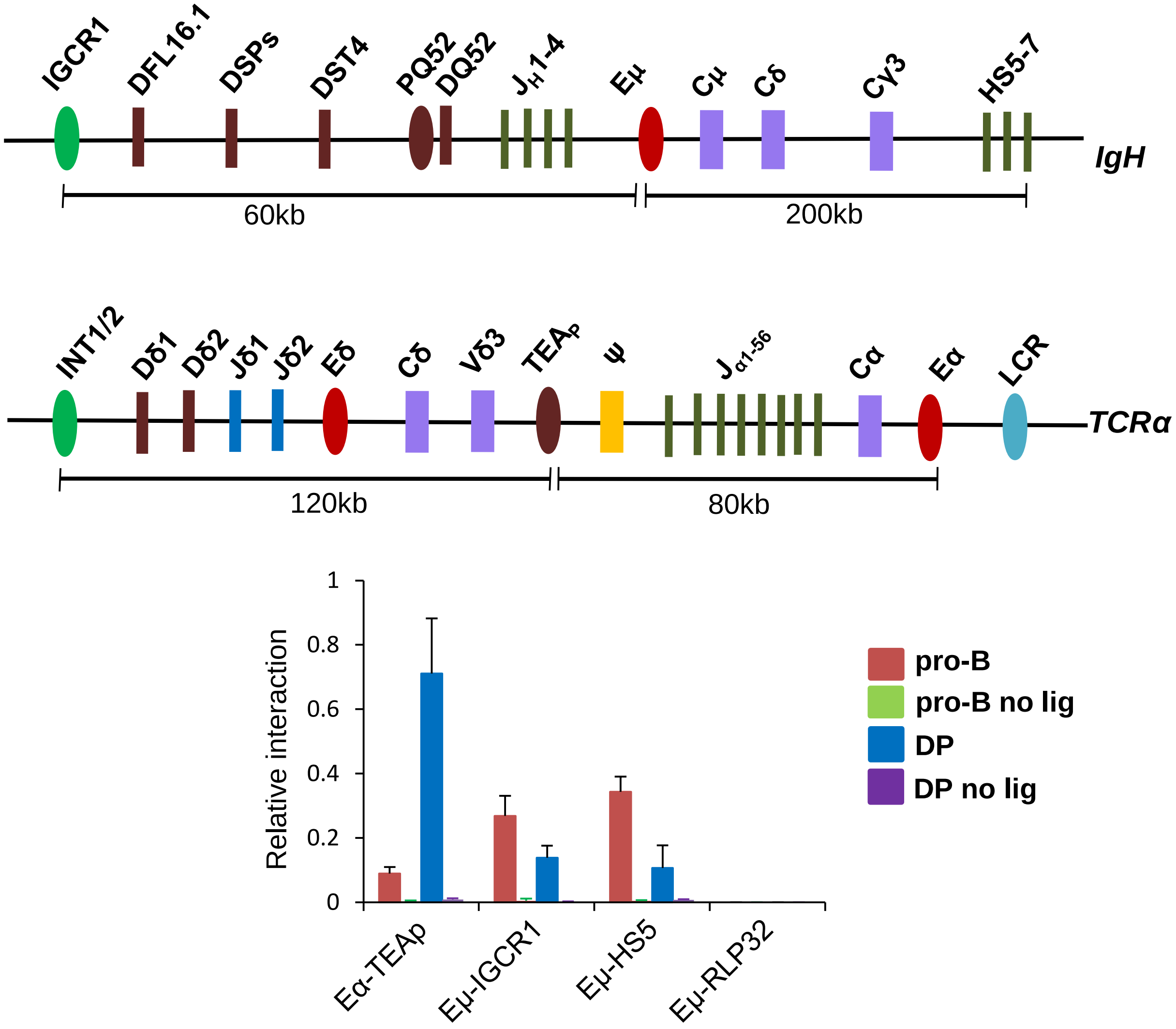

F

**Total no. of reads**	**6049515**	**7434578**
**Filtered Reads**	**2443772**	**3063534**
**Reads after duplicate removal**	**989613**	**1308846**
Gene segments	Number of reads repl1	Number of reads repl2
DSP2.9	6220	9714
DSP2.2	91717	109854
DSP2.X1	143551	169730
DSP2.X2	143201	169243
DSP2.3	23313	34264
DSP2.5	577339	759880

Figure S4

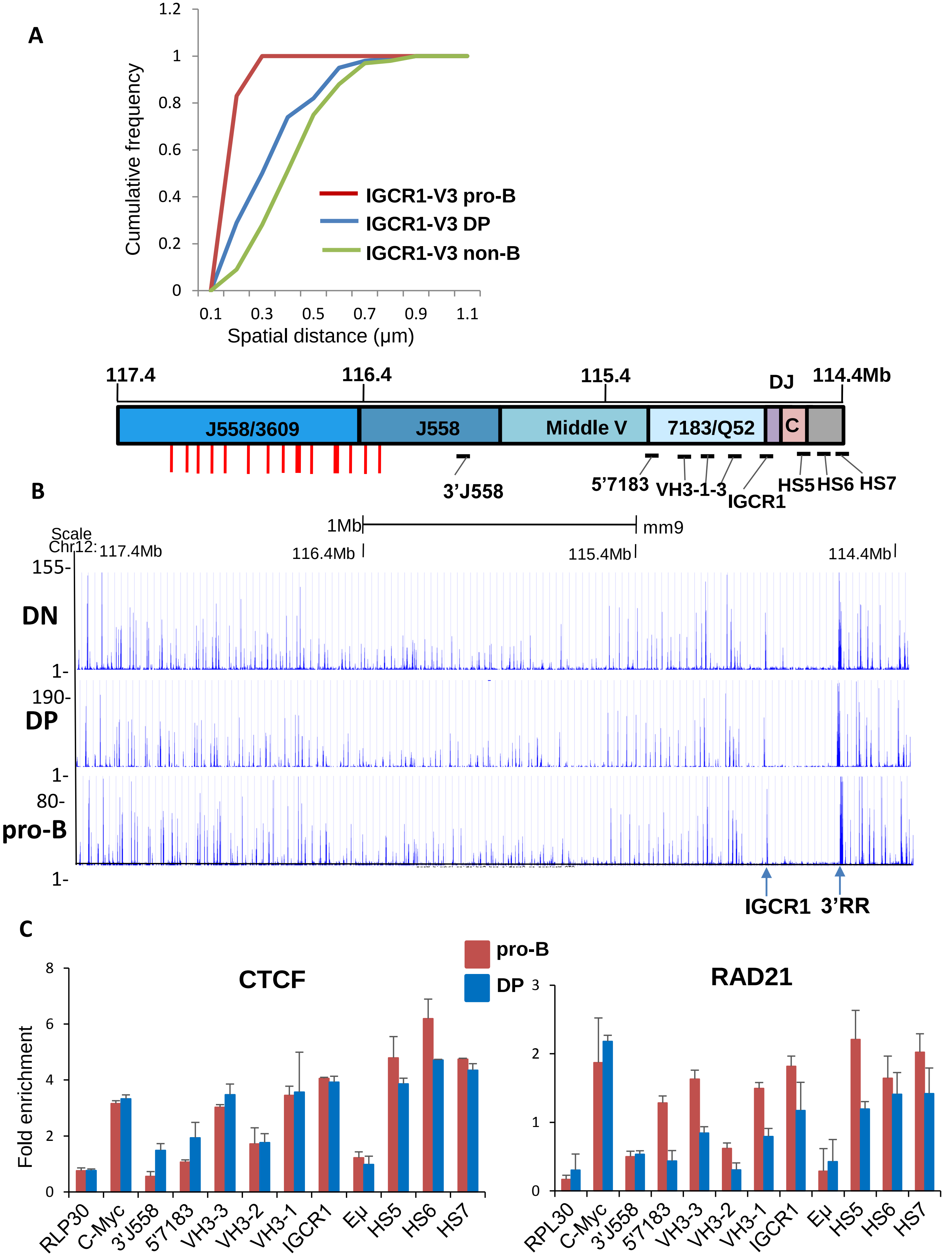
A
Cumulative frequency
1.2
1
0.8
0.6
0.4
0.2
0
0.1 0.3 0.5 0.7 0.9 1.1
Spatial distance (μm)
IGCR1-V3 pro-B
IGCR1-V3 DP
IGCR1-V3 non-B
117.4
116.4
115.4
DJ
114.4Mb
J558/3609
J558
Middle V
7183/Q52
C
3'J558
5'7183
VH3-1-3
IGCR1
HS5
HS6
HS7
B
Scale
Chr12:
1Mb
mm9
117.4Mb
116.4Mb
115.4Mb
114.4Mb
155-
DN
1-
190-
DP
1-
80-
pro-B
1-
IGCR1
3'RR
C
pro-B
DP
CTCF
Fold enrichment
8
6
4
2
0
RLP30
C-Myc
3'J558
5'7183
VH3-3
VH3-2
VH3-1
IGCR1
Eμ
HS5
HS6
HS7
RAD21
3
2
1
0
RPL30
C-Myc
3'J558
5'7183
VH3-3
VH3-2
VH3-1
IGCR1
Eμ
HS5
HS6
HS7

Figure S4

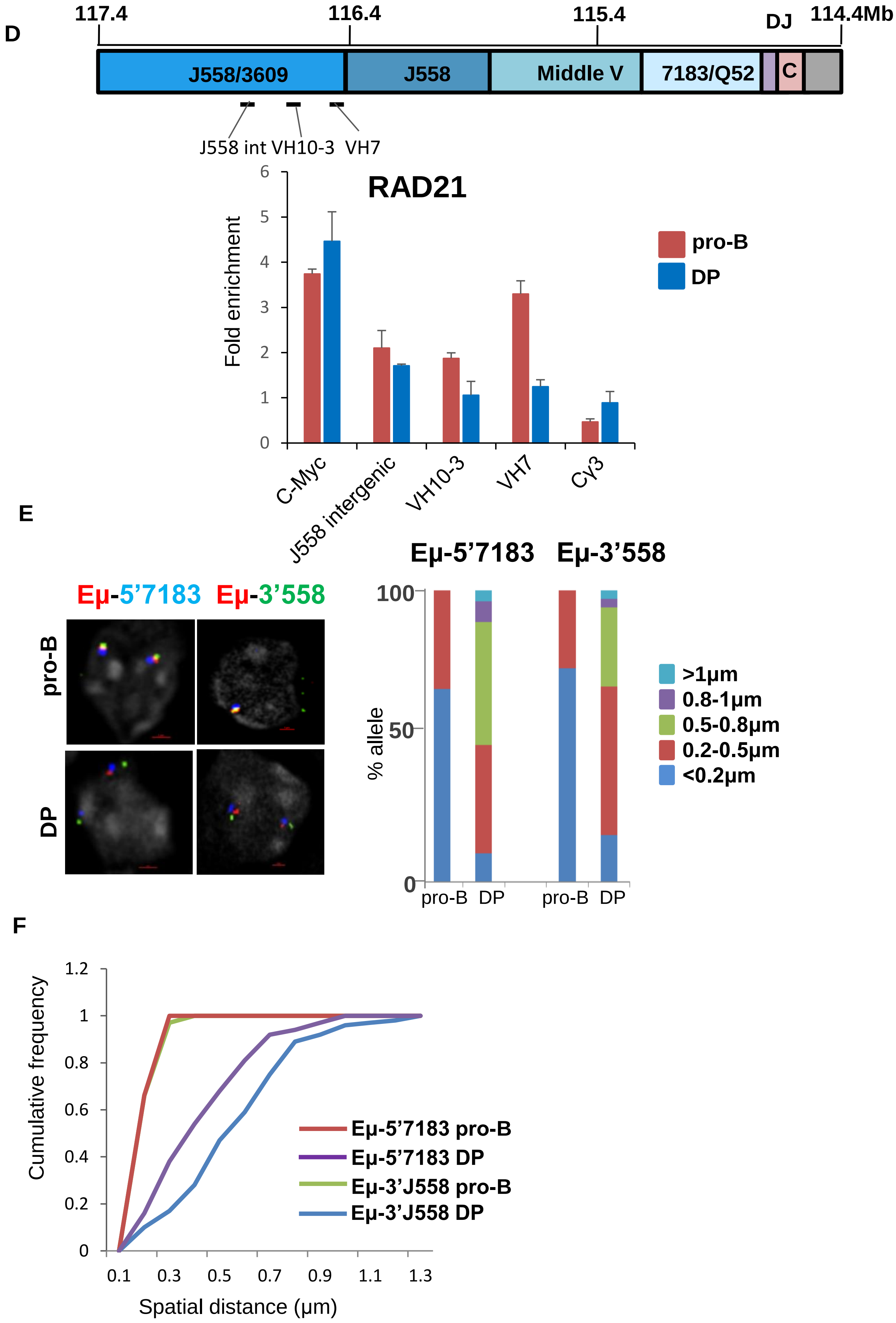

D
117.4
116.4
115.4
DJ
114.4Mb
J558/3609
J558
Middle V
7183/Q52
C
J558 int VH10-3 VH7
RAD21
Fold enrichment
pro-B
DP
C-Myc
J558 intergenic
VH10-3
VH7
Cγ3
E
Eμ-5'7183 Eμ-3'558
pro-B
DP
Eμ-5'7183 Eμ-3'558
% allele
>1μm
0.8-1μm
0.5-0.8μm
0.2-0.5μm
<0.2μm
pro-B DP pro-B DP
F
Cumulative frequency
Spatial distance (μm)
Eμ-5'7183 pro-B
Eμ-5'7183 DP
Eμ-3'J558 pro-B
Eμ-3'J558 DP
