# Supplementary material for: Misregulation of the IgH Locus in Thymocytes
Source: Front Immunol. 2018 Nov 13;9:2426. doi: 10.3389/fimmu.2018.02426 (PMC6244664; doi:10.3389/fimmu.2018.02426)
Supplement: Supplementary file 2 [file Data_Sheet_2.pdf]

Supplementary figure legends

Figure S1. Chromatin status of *IgH* locus in DP thymocytes (related to figure 1).

Top panel shows scale representation of the murine *IgH* locus based on mm9. A) $CD19^+$ pro-B cells from $Rag2^{-/-}$ and $CD4^+CD8^+$ thymocytes from TCRβ×$Rag2^{-/-}$ transgenic mice were used in chromatin immunoprecipitation using anti-H3K27me3 antibody. B) $CD19^+$ pro-B cells from $Rag2^{-/-}$ and $CD4^+CD8^+$ thymocytes derived from WT C57BL/6 mice were used in chromatin immunoprecipitation experiment using anti-H3K4me3 antibody. Amplicons were normalized to input. Position of amplicons are shown as black line below schematic. *TCF7* and *Lck* gene promoter served as positive control for DP thymocytes. Cγ3 is used as a negative control while γ-actin served as a positive control in both cell types. Results shown are the mean of two independent experiments. Y-axis shows enrichment of respective amplicons in the immunoprecipitate compared to an equal amount of input DNA as described in the methods. Error bars represent standard error of the mean (n=2).

Figure S2. Status of Eμ enhancer in DP thymocytes (related to figure 2).

A) Enhancer elements and transcription factors that bind to these sites are shown below the schematic of the D_H-C_H part of the *IgH* locus. The arrows originating from enhancer represents bidirectional transcription of eRNAs named as Iμ sense and Iμ antisense. μ0 transcripts initiate at the DQ52 promoter (PQ52) and have been shown to be Eμ-dependent. Transcription factor binding to Eμ was assayed by chromatin immunoprecipitation using anti-E2A (B) and anti-YY1 (C), anti-RUNX1 (D) and anti-HEB (E) antibodies in WT DP thymocytes. Co-precipitated DNA was quantified by qPCR and fold enrichment was calculated relative to input. Y-axis shows enrichment of respective amplicons in the immunoprecipitate relative to an equal amount of input DNA. Error bars represent standard error of the mean (n=2). IgG served as negative control.

Figure S3. D_H rearrangements in DP thymocytes (related to figure 3).

A) Rearrangement frequency of DSP2 gene segments in pro-B cells. The data is derived from VDJ-seq analysis from Choi et al. 2013 (left panel) and Bolland et al. 2016 (right panel). B) DP cells were purified from the thymus of WT C57BL/6 mice by positive selection using PE-conjugated-anti-CD8 magnetic beads. Purity of DP thymocytes was checked by CD4 and CD8 staining. C) Pro-B cells were derived from bone marrow of WT C57BL/6 mice after depletion of IgM expressing cells and other lineage cells. For pro-B cells, $B220^{int}$ and $CD43^+$ double positive cells were gated on CD19 and AA4.1. Pre-B cells were derived after gating $B220^{int}$ population on CD19 and AA4.1. D) Post-sort purity of WT pro-B cells.
E) Top panel shows locations of anchor primers and sites queried for interaction through chromosome conformation capture analyses. The region of interest is expanded on a scale view for *IgH* locus and TCRα locus. 3C analysis were done in DP thymocytes derived from TCRβ×$Rag2^{-/-}$ mice and pro-B cells derived from $Rag2^{-/-}$ mice using anchor primers located at Eμ or Eα. Relative interaction frequency was calculated after normalization for ligation efficiency using α-amylase-specific primers as described (Guo et al. 2011). The data shown here represents the mean of two independent experiments. Error bars represent standard error

of the mean (n=2). Experiment was done Relative interaction efficiency for different primer pair was calculated based on restriction digestion and re-ligation of equal moles of three bacterial artificial chromosomes (BACs) that span the region. F) Table shows total number of sequenced reads obtained for each replicate from Ion proton sequencer and number of reads aligned to each DSP2 gene segments after removal of duplicate sequence reads.

Figure S4. Status of V_H locus in DP thymocytes (related to figure 4).

A) The cumulative frequency analyses for IGCR1-V3 color-coded probe combination from figure 4A. B) Schematic of *IgH* locus is shown on top. Genome browser tracks of CTCF ChIP-Seq in pro-B cells, $CD4^+CD8^+$ (DP) and $CD4^-CD8^-$ (DN) thymocytes. ChIP-Seq track shows that CTCF binding to *IgH* locus is lymphoid-specific. CTCF ChIP-Seq was derived from (Shih et al. 2012, GSE41743). C) CTCF and RAD21 ChIP were carried out using CTCF and RAD21 antibody in pro-B cells and DP thymocytes. C-myc served as a positive control for both cell types. Cγ3 is used as a negative control. HS5-7 were used as positive control for pro-B cells. Data represents mean of two independent experiments. D) RAD21 ChIP was carried out in WT DP thymocytes (n=2). Location of primers are indicated in schematic. E) Enhancer dependent interaction was measured using FISH probes located at 3'J558, 5'7183 (V_H part of the locus) and Eμ were hybridized to pro-B and DP thymocytes. Representative nuclei are shown with probe combinations indicated on the top. Spatial distances between probes were measured after image deconvolution from 100 nuclei. F) Quantitation of FISH data is shown as percentage of *IgH* allele with spatial distances shown in figure (n=100).

Table 1. Primers list

3C		
Name	Sequence (5'-3')	References
Eμ probe	AGCTTTAAGAGCAGCAGCCACAGCT	4
Eα probe	CTGCCTGCCTGAGGACTGCCA	3
α-amylase probe	TTGAATATGTACCGAGTACACATGGATGGTGCAT	4
DFL16.1	GGATGTGAGTAGCTAGAGGATA	4
HS5	GTTTGTGTGTCTACCTTACTGTC	4
Eμ	GGAACAATTCCACACAAAGACTC	4
TEAp	CACCAACGAAAGACAAGGAC	3
α-amylase-F	GCTTCCATGATACTCTATGTTCTTCCT	4
α-amylase-R	GAGATCTTACGTAGGCACTTAGTGGTATAA	4
FISH		
Eμ-F	AGCTCATGGTACTTTGAGGAAATC	1
Eμ-R	TTGTAGGAGGACTTCCCTAATCTG	1

IGCR1-F	GTCTGGTAGAACTCTGCACTAAACCCTCTGATC	4
IGCR1-R	GCACTGTGGTAGCTACTACCGTAGTAATAAACA	4
5’7183-F	TTGGCTCACTCTGAGTTGGGATTCCTC	4
5’7183-R	TAAAGCTGAACAAGGACCACAAGACGA	4
3’558-F	AAGTCCCTGGGAGCTCTGGGGCAGTCA	4
3’558-R	TTGTTTCTAGGAAAAGATAGGCACACAGAT	4
V10-F	GCACATCTTCATTGTTCTTCTGAAATC	4
V10-R	CTGACCCAGCCTACTGAAGA GTCAAAC	4
V10-3-F	CACCTCCAATAGCACTCACAGGTTGGC	4
V10-3-R	CAAAGGCTGCCTGCACTAAGACTGGTG	4
ChIP/DNaseI/RNA		
3’J558-F	GCCAGGCTTTCTACACCTTTTCC	4
3’J558-R	CCTTGCCCTTGAACTTCTGATTG	4
5’7183-F	TTCATCCGAGACTACTCAGATCG	4
5’7183-R	AGGCTTCATGGCTGGAGAAAACA	4
VH3-1-F	TCTGAGGACACAGCCTTGTATTACT	4
VH3-1-R	GACAATTTTACAGGCTGTAACTCTG	4
VH3-2-F	GAAGGGTCGATTCACCATCTCCA	4
VH3-2-R	TCCAAGTTACTGTGCTCTCTCAGC	4
VH3-3-F	CAGAGCACCCAGGACCAGCAGGG	4
VH3-3-R	ATTTTGACGGTTTGTTGAAGATTG	4
DFL(-3)/IGCR1-F	CTAACTGTGCAATACAGAGAACTACC	4
DFL(-3)/IGCR1-R	CTACTAATAGAAATTAATGCTGGAGGG	4
DFL16.1-F	CAAAGCAGCCACCATCCAG	2
DFL16.1-R	GCAGCACGGTTGAGTTTCAG	2
DSP-F	TGTTACCTTACTTGGCAGGGATTT	2
DSP-R	TGGGTTTTTGTTGCTGGATATATC	2
DQ52-F	CCCTGTGGTCTCTGACTGGTG	6
DQ52-R	GATTTCTCAAGCCTCTCTACTTCCTC	6
JH2-F	TACTTTGACTACTGGGGC	6
JH2-R	CCCTAGTCCTTCATGACC	6
Eµ-F	GGA ATG GGA GTG AGG CTC TCT C	2
Eµ-R	CTG CAG GTG TTC TGG TTC TGA TCG G	2

Cγ3-F	TGGACAAACAGAAGTAGACATGGGTC	2
Cγ3-R	GGGGTTTAGAGGAGAGAAGGCAC	2
HS5-F	CCGCCCTTCACACCCTGACAAAC	4
HS5-R	CTGGCACTGAGCAAGCAAACTCT	4
HS6-F	AGCAGAGGTTGCAGTGGTGCATC	4
HS6-R	ACTTCCCTGTGGGCTTTGAGTTT	4
HS7-F	GAATGGGCAGATGAACTTGGGTC	4
HS7-R	TCATGGCAGTGTCCAGTCAACAC	4
Eα-F	AGG AAG TCG CAG AAC CTG AA	3
Eα-R	GAG GGA GAA AGC CTT TTG GT	3
Eβ-F	GGGGGAAGGGGTGGAAGCATCTCACC	
Eβ-R	AGG ACC TGG TAA ATG TCA AA	
TCF7-F	TTTCCTTGTGTGTGCGAGAG	
TCF7-R	ATCTTTTCCGTTGCCCAGTT	
LCK-F	CACCAGACTGGCCTTGAACT	
LCK-R	GTTTGACTGGGATGGAGGAA	
CD79a-F	CCACGCACTAGAGAGAGACTCAA	5
CD79a-R	CCGCCTCACTTCCTGTTCAGCCG	5
RPL32-F	AGTTTTCTTTAGAGGACCCAGAG	
RPL32-R	AGGCAGCGCCGAGGAAGAAGTGG	
RPL30-F	AGCAACCAACTACCGCAGACTACT	8
RPL30-R	ATCCAGAGCGTCAAACACCAGCTA	8
Myc-F	AAGGAAGCATCTTCCCAGAAC	3
Myc-R	AAGTGTGCCCTCTACTGGCCA	3
Ccnd3-F	TCGAGGCCATTCCTAGAAGCCA	
Ccnd3-R	GCCAAAAGTTATTCCTTCGTG	
Imu-F	AGTTTAACCGAGGAATGGGAGTGAG	
Imu-R	GTTTGGTGGGGCTGGACAGAGTGTTT	
Sense-F	AGGGCTCTCAACCTTGTTCC	
Sense-R	TAGGCCTGGACTTTGGGTCT	
Antisense-F	TTTTCCCTTCCCCAAATAGC	
Antisense-R	GGGTCAAGGAACCTCAGTCA	
Imu-sense	AGGCAGCCACAGCTGTGGCTGCTGCTCT	

Imu antisense	TGCTTTTTAGAGCCTCGCTTACTAGGGCT	
Lck-F	GAAGCCTTCTTGGCCAGTC	
Lck-R	GGAGACTTGGGCTTTGAGAA	
J558 intergenic F	CTGCAGTGCAGATCAGTTAGTA	
J558 intergenic R	TAAGCCAGACAATGTAACTTCAG	
V10-3F	CAGCATCTCTCTGCTGACCA	4
V10-3R	CTAGTCAAGTCAGACTGGGCAAC	4
VH7F	CATACACAGCATCTCTCTGCTGACA	7
VH7R	AACTCATACAACTCAAGTCAGAC	7
DJ recombination assay		
DFL16.1-F	ACA CCT GCA AAA CCA GAG ACC ATA	2
DSP-F	ATG GCC CCT GAC ACT CTG CAC TGC TA	2
DQ52-F	GCGACTGTTTTGAGAGAAATCATTGG	2
J_H4-R	GGGTCTAGACTCTCAGCCGGCTCCCTCAGGG	2
β-globin-F	GCC TTG CCT GTT CCT GCT C	2
β-globin -R	ATT GAG CCC TTT ACT CTC TCT GTT C	2
J_H1-R	TGAGGAGACGGTGACCGTGGTCCC	1

References

1. Qiu X, Kumari G, Gerasimova T, Du H, Labaran L, Singh A, De S, Wood WH 3rd, Becker KG, Zhou W, Ji H, Sen R. 2018.Sequential Enhancer Sequestration Dysregulates Recombination Center Formation at the IgH Locus. Molecular Cell 70: 21-33.

2. Subrahmanyam R, Du H, Ivanova I, Chakraborty T, Ji Y, Zhang Y, Alt FW, Schatz DG, Sen R. 2012. Localized epigenetic changes induced by DH recombination restricts recombinase to DJH junctions. Nature Immunology 12: 1205-12.

3. Shih HY, Verma-Gaur J, Torkamani A, Feeney AJ, Galjart N, Krangel MS. 2012.Tcra gene recombination is supported by a Tcra enhancer- and CTCF-dependent chromatin hub. Proc Natl Acad Sci U S A. 109: E3493-502.

4. Guo C, Gerasimova T, Hao H, Ivanova I, Chakraborty T, Selimyan R, Oltz EM, Sen R. 2011. 1.Two forms of loops generate the chromatin conformation of the immunoglobulin heavy-chain gene locus.Cell. 147:332-43.

5. Lin YC, Jhunjhunwala S, Benner C, Heinz S, Welinder E, Mansson R, Sigvardsson M, Hagman J, Espinoza CA, Dutkowski J, Ideker T, Glass CK, Murre C. 2010. A global network of transcription factors, involving E2A, EBF1 and Foxo1, that orchestrates B cell fate. Nature Immunology. 11:635-43.

6. Chakraborty T, Perlot T, Subrahmanyam R, Jani A, Goff PH, Zhang Y, Ivanova I, Alt FW, Sen R. 2009. A 220-nucleotide deletion of the intronic enhancer reveals an epigenetic hierarchy in immunoglobulin heavy chain locus activation. J Exp Med. 206:1019-27.

7. Degner SC, Wong TP, Jankevicius G, Feeney AJ, 2009: Cutting Edge: Developmental Stage-Specific Recruitment of Cohesin to CTCF Sites throughout Immunoglobulin Loci during B Lymphocyte Development. J. Immunology. 182: 44-48.

8. Liu H, Schmidt-Supprian M, Shi Y, Hobeika E, Barteneva N, Jumaa H, Pelanda R, Reth M, Skok J, Rajewsky K, Shi Y. 2007. Yin Yang 1 is a critical regulator of B-cell development. Genes & Develoment 21:1179-89.
