# Supplementary material for: Misregulation of the IgH Locus in Thymocytes
Source: Front Immunol. 2018 Nov 13;9:2426. doi: 10.3389/fimmu.2018.02426 (PMC6244664; doi:10.3389/fimmu.2018.02426)
Supplement: Supplementary file 3 [file Data_Sheet_3.pdf]

Table S2: Sequences of recombined product derived from DJ-seq data

Germline DSP2.9	Heptamer	Heptamer	J_H1
TACTGTGTCTATGATGGTTACTAC	CACAGTG	CACTGTG	ACTGGTACTTCGATGTCTGGGGCGCAGGGACCACGGTCACCGTCTCCTC

**DSP2.9-J_H1**	**N addition**	
TACTGTGTCTATGATGGTTACTA-	GTGACCA	---CTGGTACTTCGATGTCTGGGGCGCAGGGACCACGGTCACCGTCTCCTC
TACTGTGTCTATGATGGTTACT--	GGGTAC	-----TGGTACTTCGATGTCTGGGGCGCAGGGACCACGGTCACCGTCTCCTC
TACTGTGTCTATGATGGTTACTAC	CTAGGGGACTA	--------GTACTTCGATGTCTGGGGCGCAGGGACCACGGTCACCGTCTCCTC
TACTGTGTCTATGATGGTTAC-----	GGTAAGCTC	----------TACTTCGATGTCTGGGGCGCAGGGACCACGGTCACCGTCTCCTC
TACTGTGTCTATGATGGTTACTA-	GTAAGCTC	-------------CTTCGATGTCTGGGGCGCAGGGACCACGGTCACCGTCTCCTC
TACTGTGTCTATGATGGTTAC---	GTAAGCTAC	------------CTTCGATGTCTGGGGCGCAGGGACCACGGTCACCGTCTCCTC
TACTGTGTCTATGATGGTTACT--	GCCTTCA	----------TACTTCGATGTCTGGGGCGCAGGGACCACGGTCACCGTCTCCTC

Germline DSP2.2	Heptamer	Heptamer	J_H1
TACTGTGTCTACTATGATTACGAC	CACAGTG	CACTGTG	ACTGGTACTTCGATGTCTGGGGCGCAGGGACCACGGTCACCGTCTCCTC

**DSP2.2-J_H1**	**N addition**	
TACTGTGTCTACTATGATTAC----	CAGGATGA	----GTACTTCGATGTCTGGGGCGCAGGGACCACGGTCACCGTCTCCTC
TACTGTGTCTACTATGATTACGAC	TTAGAAGGAT	----------CTTCGATGTCTGGGGCGCAGGGACCACGGTCACCGTCTCCTC
TACTGTGTCTACTATGATTACG---	GGACGGTT	----GTACTTCGATGTCTGGGGCGCAGGGACCACGGTCACCGTCTCCTC
TACTGTGTCTACTATGATTACG --	GACGCAGA	-----------------GATGTCTGGGGCGCAGGGACCACGGTCACCGTCTCCTC
TACTGTGTCTACTATGATTACGA -	GGAATTGT	-----------------GATGTCTGGGGCGCAGGGACCACGGTCACCGTCTCCTC
TACTGTGTCTACTATGATTACG --	GCCCTTGACC	-------------TTCGATGTCTGGGGCGCAGGGACCACGGTCACCGTCTCCTC
TACTGTGTCTACTATGATTACG--	GTCCTTAC	-------ACTTCGATGTTCTGGGGCGCAGGGACCACGGTCACCGTCTCCTC
TACTGTGTCTACTATGATTACGAC	CACAGTG	-----------TTCGATGTCTGGGGCGCAGGGACCACGGTCACCGTCTCCTC
TACTGTGTCTACTATGATTACG---	CATGTAGCAG	-------ACTTCGATGTCTGGGGCGCAGGGACCACGGTCACCGTCTCCTC

Germline DSP2.x1	Heptamer	Heptamer	J_H1
TACTGTGCCTACTATAGTAACTAC	CACAGTG	CACTGTG	ACTGGTACTTCGATGTCTGGGGCGCAGGGACCACGGTCACCGTCTCCTC

**DSP2.x1-J_H1**	**N addition**	
TACTGTGCCTACTATAGTAACTA-	TGGCGGCC	----------CTTCGATGTCTGGGGCGCAGGGACCACGGTCACCGTCTCCTC
TACTGTGCCTACTATAGTAACTA-	GTACATT	--TGGTACTTCGATGTCTGGGGCGCAGGGACCACGGTCACCGTCTCCTC
TACTGTGCCTACTATAGTAACTAC	GTCCTTACGGT	-----GGTACTTCGATGTCTGGGGCGCAGGGACCACGGTCACCGTCTCCTC
TACTGTGCCTACTATAGTAACTA--	GAGCTTGA	-----GGTACTTCGATGTCTGGGGCGCAGGGACCACGGTCACCGTCTCCTC
TACTGTGCCTACTATAGTAACT---	CCTACGTC	------GTACTTCGATGTCTGGGGCGCAGGGACCACGGTCACCGTCTCCTC
TACTGTGCCTACTATAGTAACTA	CCCTACAC	--------------TTCGATGTCTGGGGCGCAGGGACCACGGTCACCGTCTCCTC
TACTGTGCCTACTATAGTAAC---	GGGTACGT	---------TACTTCGATTGTCTGGGGCGCAGGGACCACGGTCACCGTCTCCTC
TACTGTGCCTACTATAGTAACTA-	GGGACCTT	----------------TTCGATGTCTGGGGCGCAGGGACCACGGTCACCGTCTCCTC
TACTGTGCCTACTATAGTAAC----	GTAAGGCCATG	------------ACTTCGATGTCTGGGGCGCAGGGACCACGGTCACCGTCTCCTC
TACTGTGCCTACTATAGTAACTAC	TGGGGCC	---CTGGTACTTCGATGTCTGGGGCGCAGGGACCACGGTCACCGTCTCCTC

Germline	DSP2.x2	Heptamer	Heptamer	J_H1
	TACTGTGCCTACTATAGTAACTAC	CACAGTG	CACTGTG	ACTGGTACTTCGATGTCTGGGGCGCAGGGACCACGGTCACCGTCTCCTC

**DSP2.x2-J_H1**	**N addition**	
TACTGTGCCTACTATAGTAAC--	TTGGCCCC	----GGTACTTCGATGTCTGTGGCGCAGGGACCACGGTCACCGTCTCCTC
TACTGTGCCTACTATAGTAACTA-	TTGGCCCC	------ACTTCGATGTCTGGGGCGCAGGGACCACGGTCACCGTCTCCTC
TACTGTGCCTACTATAGTAACTAC	CCTGGTT	-TGGTACTTCGATGTCTGGGGCGCAGGGACCACGGTCACCGTCTCCTC
TACTGTGCCTACTATAGTAACTA-	GACCCGTA	----------------ATGTCTGGGGCGCAGGGACCACGGTCACCGTCTCCTC
TACTGTGCCTACTATAGTAACTA-	ACCTTTG	-----TACTTCGATGTCTGGGGCGCAGGGACCACGGTCACCGTCTCCTC
TACTGTGCCTACTATAGTAACTAC	GGATTCTGG	----GTACTTCGATGTCTGGGGCGCAGGGACCACGGTCACCGTCTCCTC
TACTGTGCCTACTATAGTAACT--	ACTACTGGG	---TGGTACTTCGATTCTGGGGCGCAGGGACCACGGTCACCGTCTCCTC
TACTGTGCCTACTATAGTAACT --	ACTGCTGTAC	----ACTTCGATGGGTCTGGGGCGCAGGGACCACGGTCACCGTCTCCTC
TACTGTGCCTACTATAGTAAC---	ATTGCGTAG	-------CTTCGATGTCTTTGGGGCGCAGGGACCACGGTCACCGTCTCCTC
TACTGTGCCTACTATAGTAA----	CCGGTAGCGTA	------------------TGTCCTGGGGCGCAGGGACCACGGTCACCGTCTCCTC

Germline	DSP2.3/4	Heptamer	Heptamer	J_H1
	TACTGTGTCTACTATGGTTACGAC	CACAGTG	CACTGTG	ACTGGTACTTCGATGTCTGGGGCGCAGGGACCACGGTCACCGTCTCCTC

**DSP2.3/4-J_H1**	**N addition**	
TACTACTGTGTCTACTATGGTTACG	GGGCTA	--------TACTTCGATGTCTGGGGCGCAGGGACCACGGTCACCGTCTCCTC
TACTACTGTGTCTACTATGGTTAC-	GTCCTTC	--------TACTTCGATGTCTGGGGCGCAGGGACCACGGTCACCGTCTCCTC
TACTACTGTGTCTACTATGGTTACG	GCCGGATG	------TACTTCGATGTCTGGGGCGCAGGGACCACGGTCACCGTCTCCTC
TACTACTGTGTCTACTATGGTTA--	AGGACTTCTCC	---------CTTCGATGTCTGGGGCGCAGGGACCACGGTCACCGTCTCCTC
TACTACTGTGTCTACTATGGTTAC-	TGGGG	-----TACTTCGATGTCTGGGGCGCAGGGACCACGGTCACCGTCTCCTC
TACTACTGTGTCTACTATGGTTA--	GTCCTTG	---------------CGTGTCTGGGGCGCAGGGACCACGGTCACCGTCTCCTC

Germline	DSP2.5	Heptamer	Heptamer	J_H1
	TACGTGTCTACTATGGTAACTAC	CACAGTG	CACTGTG	ACTGGTACTTCGATGTCTGGGGCGCAGGGACCACGGTCACCGTCTCCTC

**DSP2.5-J_H1**	**N addition**	
TACTGTGTCTACTATGGTAACT--	GGGGTC	--------- TACTTGATGTCTGGGGCGCAGGGACCACGGTCACCGTCTCCTC
TACTGTGTCTACTATGGTAAC---	TGCGGTGA	------GTACTTCGATGTCTGGGGCGCAGGGACCACGGTCACCGTCTCCTC
TACTGTGTCTACTATGGTAACTAC	TTTAGCTAC	-------------CGATGTCTGGGGCGCAGGGACCACGGTCACCGTCTCCTC
TACTGTGTCTACTATGGTAACT--	GTCTGAGT	----------TTCGATGTCTGGGGCGCAGGGACCACGGTCACCGTCTCCTC
TACTGTGTCTACTATGGTAACTA-	GGACCC	-------TTCGATGTCTGGGGCGCAGGGACCACGGTCACCGTCTCCTC
TACTGTGTCTACTATGGTAAC---	GACCCTG	----------------GATGTCTGGGGCGCAGGGACCACGGTCACCGTCTCCTC
TACTGTGTCTACTATGGTAACTAC	GTTACGTCTT	----- ACTTCGATGTCTGGGGCGCAGGGACCACGGTCACCGTCTCCTC
TACTGTGTCTACTATGGTAACT--	GGATGCCT	---TGGTACTTCGATGTCTGGGGCGCAGGGACCACGGTCACCGTCTCCTC
TACTGTGTCTACTATGGTAACT--	CTCGAGC	------------CTTCGATGTCTGGGGCGCAGGGACCACGGTCACCGTCTCCTC
TACTGTGTCTACTATGGTAACTA-	CTCCGTGAG	------------CTTCGATGTCTGGGGCGCAGGGACCACGGTCACCGTCTCCTC

Germline DSP2.5 Heptamer Heptamer J_H1

TACGTGTCTACTATGGTAACTAC CACAGTG ______ CACTGTG ACTGGTACTTCGATGTCTGGGGCGCAGGGACCACGGTCACCGTCTCCTC

**DSP2.5-J_H1**	**N addition**	
TACTGTGTCTACTATGGTAACTA-	TACCG	------TACTTCGATGTCTGGGGCGCAGGGACCACGGTCACCGTCTCCTC
TACTGTGTCTACTATGGTAACT--	GAGCTTGG	--------ACTTCGATGTCTGGGGCGCAGGGACCACGGTCACCGTCTCCTC
TACTGTGTCTACTATGGTAACT--	TCCATGA	--------ACTTCGATGTCTGGGGCGCAGGGACCACGGTCACCGTCTCCTC
TACTGTGTCTACTATGGTAACTA-	GCCGCTAGG	---------CTTCGATGTCTGGGGCGCAGGGACCACGGTCACCGTCTCCTC
TACTGTGTCTACTATGGTAACTA-	TAAAGCCTG	--------ACTTCGATGTCTGGGGCGCAGGGACCACGGTCACCGTCTCCTC
TACTGTGTCTACTATGGTAAC---	CCTGCTA	--------------GATGTCTGGGGCGCAGGGACCACGGTCACCGTCTCCTC
TACTGTGTCTACTATGGTAACT--	GACCTGT	------TACTTCGATGTCTGGGGCGCAGGGACCACGGTCACCGTCTCCTC
TACTGTGTCTACTATGGTAACT--	CCTGCTA	----------CTTCGATGTCTGGGGCGCAGGGACCACGGTCACCGTCTCCTC
TACTGTGTCTACTATGGTAACTA-	CGTGCCAGC	------------CTTCGATGTCTGGGGCGCAGGGACCACGGTCACCGTCTCCTC
TACTGTGTCTACTATGGTAACT--	CCCCT	--------------CGATGTCTGGGGCGCAGGGACCACGGTCACCGTCTCCTC
TACTGTGTCTACTATGGTAAC---	CGACGGG	--TGGTACTTCGATGTCTGGGGCGCAGGGACCACGGTCACCGTCTCCTC
TACTGTGTCTACTATGGTAACTAC	TAAG	------TACTTCGATGTCTGGGGCGCAGGGACCACGGTCACCGTCTCCTC
TACTGTGTCTACTATGGTAACTA-	GGCTA	------TACTTCGATGTCTGGGGCGCAGGGACCACGGTCACCGTCTCCTC
TACTGTGTCTACTATGGTAACT--	GGAACAGTG	-----GGTACTTCGATGCTGGGGCGCAGGGACCACGGTCACCGTCTCCTC
TACTGTGTCTACTATGGTAAC---	CCCTACC	----------------CGATGTCTGGGGCGCAGGGACCACGGTCACCGTCTCCTC
TACTGTGTCTACTATGGTAACT--	GAGTCTTGC	------GTACTTCGATGCTGGGGCGCAGGGACCACGGTCACCGTCTCCTC
TACTGTGTCTACTATGGTAAC--	GAGTCCTCAC	-----------TCGATGTCTGGGGCGCAGGGACCACGGTCACCGTCTCCTC
TACTGTGTCTACTATGGTAACT--	CCCTGTATCG	------ACTTCGATGTCTGGGGCGCAGGGACCACGGTCACCGTCTCCTC
TACTGTGTCTACTATGGTAAC---	TCCAGCCTGA	---------TCGATGTCTGGGGCGCAGGGACCACGGTCACCGTCTCCTC
TACTGTGTCTACTATGGTAA----	GTCGGTACTG	--GGTACTTCGATGTCTGGGGCGCAGGGACCACGGTCACCGTCTCCTC
TACTGTGTCTACTATGGTAAC---	GAGCCTTGC	------GTACTTCGATGTCTGGGGCGCAGGGACCACGGTCACCGTCTCCTC
TACTGTGTCTACTATGGTAAC--	GACTCCTTACC	------GTACTTCGATGTCTGGGGCGCAGGGACCACGGTCACCGTCTCCTC
TACTGTGTCTACTATGGTAA----	CCTGGGTCG	------ACTTCGATGTCTGGGGCGCAGGGACCACGGTCACCGTCTCCTC
TACTGTGTCTACTATGGTAAC---	GCCAGCGTTG	---------CTTCGATGTCTGGGGCGCAGGGACCACGGTCACCGTCTCCTC
TACTGTGTCTACTATGGTAAC----	TCGACCCTG	------TACTTCGATGTCTGGGGCGCAGGGACCACGGTCACCGTCTCCTC
TACTGTGTCTACTATGGTAACT--	CCAGCGCCG	-----------TTCGATGTCTGGGGCGCAGGGACCACGGTCACCGTCTCCTC
TACTGTGTCTACTATGGTAACTA-	CCGACCCAG	------TACTTCGATGTCTGGGGCGCAGGGACCACGGTCACCGTCTCCTC

Table S3: Sequences of recombined product from cloning

Germline DSP2.9	Heptamer	Heptamer	J_H1
TACTGTGTCTATGATGGTTACTAC	CACAGTG	CACTGTG	ACTGGTACTTCGATGTCTGGGGCGCAGGGACCACGGTCACCGTCTCCTC

DSP2.9-J_H1	N addition	
TACTGTGTCTATGATGGTTACT--	GCTGGTG	---------TACTTCGATGTCTGGGGCGCAGGGACCACGGTCACCGTCTCCTC
TACTGTGTCTATGATGGTTACT--	AGACTCTGC	------GTACTTCGATGTCTGGGGCGCAGGGACCACGGTCACCGTCTCCTC
TACTGTGTCTATGATGGTTACTA-	GATTCTG	-CTGGTACTTCGATGTCTGGGGCGCAGGGACCACGGTCACCGTCTCCTC
TACTGTGTCTATGATGGTTAC-----	CTCGAGTGA	-----------CTTCGATGCTGGGGCGCAGGGACCACGGTCACCGTCTCCTC

Germline DSP2.2	Heptamer	Heptamer	J_H1
TACTGTGTCTACTATGATTACGAC	CACAGTG	CACTGTG	ACTGGTACTTCGATGTCTGGGGCGCAGGGACCACGGTCACCGTCTCCTC

DSP2.2-J_H1	N addition	
TACTGTGTCTACTATGATTACG---	GGGGCG	--ACTGGTACTTCGATGTCTGGGGCGCAGGGACCACGGTCACCGTCTCCTC
TACTGTGTCTACTATGATTACGA-	CAGTGTCT	-----GGTACTCGATGTCTGGGGCGCAGGGACCACGGTCACCGTCTCCTC
TACTGTGTCTACTATGATTAC----	CGGGTGTC	--------TACTTCGATGTCTGGGGCGCAGGGACCACGGTCACCGTCTCCTC
TACTGTGTCTACTATGATTAC---	GGGTTGAC	----TGGTACTTCGATGTCTGGGGCGCAGGGACCACGGTCACCGTCTCCTC

Germline DSP2.x1	Heptamer	Heptamer	J_H1
TACTGTGCCTACTATAGTAACTAC	CACAGTG	CACTGTG	ACTGGTACTTCGATGTCTGGGGCGCAGGGACCACGGTCACCGTCTCCTC

DSP2.x1-J_H1	N addition	
TACTGTGCCTACTATAGTAACT--	CAGAGTTAGCA	--------GTACTTCGATGTCTGGGGCGCAGGGACCACGGTCACCGTCTCCTC
TACTGTGCCTACTATAGTAACTA-	AAGCCTGG	----TGGTACTTCGATGTCTGGGGCGCAGGGACCACGGTCACCGTCTCCTC
TACTGTGCCTACTATAGTAACTA-	CGTTGCCAGC	---------TACTT-CGATGTCTGGGGCGCAGGGACCACGGTCACCGTCTCCTC

Germline DSP2.x2	Heptamer	Heptamer	J_H1
TACTGTGCCTACTATAGTAACTAC	CACAGTG	CACTGTG	ACTGGTACTTCGATGTCTGGGGCGCAGGGACCACGGTCACCGTCTCCTC

DSP2.x2-J_H1	N addition	
TACTGTGCCTACTATAGTAACTA-=-	CTTGGTCCT	-------GGTACTTCGATGTCTGTGGCGCAGGGACCACGGTCACCGTCTCCTC
TACTGTGCCTACTATAGTAACTAC	TCCCCT	----CTGGTACTTCGATGTCTGGGGCGCAGGGACCACGGTCACCGTCTCCTC
TACTGTGCCTACTATAGTAACTAC	TCTGAGCT	---------GGTACTTCGATGTCTGGGGCGCAGGGACCACGGTCACCGTCTCCTC
TACTGTGCCTACTATAGTAACTA-	GTGAGTCC	--------------TACTCGATGTCTGGGGCGCAGGGACCACGGTCACCGTCTCCTC

Germline DSP2.3/4	Heptamer	Heptamer	J_H1
TACTGTGTCTACTATGGTTACGAC	CACAGTG	CACTGTG	ACTGGTACTTCGATGTCTGGGGCGCAGGGACCACGGTCACCGTCTCCTC

DSP2.3/4-J_H1	N addition	
TACTACTGTGTCTACTATGGTTAC-	GGCGTAG	---CTGGTACTTCGATGTCTGGGGCGCAGGGACCACGGTCACCGTCTCCTC
TACTACTGTGTCTACTATGGTTA--	TGGCAGTGA	------------TACTTCGATGTCTGGGGCGCAGGGACCACGGTCACCGTCTCCTC

Germline DSP2.5 Heptamer Heptamer J_H1

TACGTGTCTACTATGGTAACTACCACAGTG CACTGTG ACTGGTACTTCGATGTCTGGGGCGCAGGGACCACGGTCACCGTCTCCTC

**DSP2.5-J_H1**	**N addition**	
TACTGTGTCTACTATGGTAAC---	GTGGCCT	-------- GGTACTTGATGTCTGGGGCGCAGGGACCACGGTCACCGTCTCCTC
TACTGTGTCTACTATGGTAAC---	CCTTTGA	------TGGTACTTCGATGTCTGGGGCGCAGGGACCACGGTCACCGTCTCCTC
TACTGTGTCTACTATGGTAACTAC	ATTCTGAAC	------------------ACTTCGTCTGGGGCGCAGGGACCACGGTCACCGTCTCCTC
TACTGTGTCTACTATGGTAACTA--	TGAGTCCT	--------------ACTTCGATGTCTGGGGCGCAGGGACCACGGTCACCGTCTCCTC
TACTGTGTCTACTATGGTAACT--	TGTCGTGC	----------------TCGATGTCCCTGGGGCGCAGGGACCACGGTCACCGTCTCCTC
TACTGTGTCTACTATGGTAA----	CCGATG	--------------ACTTCGATGTCTGGGGCGCAGGGACCACGGTCACCGTCTCCTC
TACTGTGTCTACTATGGTAACTA-	CCGTGG	--------------ACTT CGTTGTCTGGGGCGCAGGGACCACGGTCACCGTCTCCTC
TACTGTGTCTACTATGGTAAC---	GTCACTA	------------TACTTCGATGTCTTGGGGCGCAGGGACCACGGTCACCGTCTCCTC
TACTGTGTCTACTATGGTAACTA--	GTGCCG	--------------ACTTCGATGTCTGGGGCGCAGGGACCACGGTCACCGTCTCCTC
TACTGTGTCTACTATGGTAACT--	GGATTCTG	--------------------CGATGTCTGGGGCGCAGGGACCACGGTCACCGTCTCCTC
TACTGTGTCTACTATGGTAACTA--	GGCATC	-------------ACTTCGATGTCTGGGGCGCAGGGACCACGGTCACCGTCTCCTC
TACTGTGTCTACTATGGTAACTAC	GGGGCTTA	----------GTACTTCGATGTCTGGGGCGCAGGGACCACGGTCACCGTCTCCTC
TACTGTGTCTACTATGGTAACT--	GATTCTG	------------TACTTCGATGTCTGGGGCGCAGGGACCACGGTCACCGTCTCCTC
TACTGTGTCTACTATGGTAACT--	AGGGCTC	--------GGTACTTCGATGTCTGGGGCGCAGGGACCACGGTCACCGTCTCCTC
TACTGTGTCTACTATGGTAA----	CGTAACA	--------------------TTCGATGTCTGGGGCGCAGGGACCACGGTCACCGTCTCCTC
TACTGTGTCTACTATGGTAACTA--	GCGGTCCC	----------GGTACTTCGATGTCTGGGGCGCAGGGACCACGGTCACCGTCTCCTC
TACTGTGTCTACTATGGTAAC---	GGTGA	---------TGGTACTTCGATGTCTGGGGCGCAGGGACCACGGTCACCGTCTCCTC
TACTGTGTCTACTATGGTAACT--	GTCCTTG	----------TGGTACTTCGATGTCTGGGGCGCAGGGACCACGGTCACCGTCTCCTC
TACTGTGTCTACTATGGTAACTA-	GGTAGCAG	------------GTACTTCGATGTCTGGGGCGCAGGGACCACGGTCACCGTCTCCTC
